# Ubiquitous Order‐Disorder Transition in the Mn Antisite Sublattice of the (MnBi_2_Te_4_)(Bi_2_Te_3_)_*n*_ Magnetic Topological Insulators

**DOI:** 10.1002/advs.202402753

**Published:** 2024-07-08

**Authors:** Manaswini Sahoo, Ifeanyi John Onuorah, Laura Christina Folkers, Ekaterina Kochetkova, Evgueni V. Chulkov, Mikhail M. Otrokov, Ziya S. Aliev, Imamaddin R. Amiraslanov, Anja U. B. Wolter, Bernd Büchner, Laura Teresa Corredor, Chennan Wang, Zaher Salman, Anna Isaeva, Roberto De Renzi, Giuseppe Allodi

**Affiliations:** ^1^ Leibniz IFW Dresden Helmholtzstraße 20 D‐01069 Dresden Germany; ^2^ Institut für Festkörper‐ und Materialphysik Technische Universität Dresden 01062 Dresden Germany; ^3^ Würzburg‐Dresden Cluster of Excellence ct.qmat Dresden Germany; ^4^ Dipartimento di Scienze Matematiche Fisiche e Informatiche Universitá di Parma Parco delle Scienze 7A Parma I‐43124 Italy; ^5^ Van der Waals‐Zeeman Institute Department of Physics and Astronomy University of Amsterdam Science Park 094 Amsterdam 1098 XH Netherlands; ^6^ Donostia International Physics Center Sebastián 20018 Donostia‐San Spain; ^7^ Departamento de Polímeros y Materiales Avanzados: Física, Química y Tecnología, Facultad de Ciencias Químicas Universidad del País Vasco UPV/EHU Donostia‐San Sebastián 20018 Spain; ^8^ Centro de Física de Materiales (CFM‐MPC) Centro Mixto (CSIC‐UPV/EHU) Donostia‐San Sebastián 20018 Spain; ^9^ Saint Petersburg State University Saint Petersburg 199034 Russia; ^10^ Instituto de Nanociencia y Materiales de Aragón (INMA) CSIC‐Universidad de Zaragoza Zaragoza 50009 Spain; ^11^ Baku State University Baku AZ1148 Azerbaijan; ^12^ Institute of Physics Ministry of Science and Education Republic of Azerbaijan Baku AZ1143 Azerbaijan; ^13^ Laboratory for Muon Spin Spectroscopy Paul‐Scherrer‐Institute Villigen PSI CH‐5232 Switzerland; ^14^ Faculty of Physics Technical University of Dortmund Otto‐Hahn‐Str. 4 44221 Dortmund Germany; ^15^ Research Center Future Energy Materials and Systems (RC FEMS) Germany

**Keywords:** cation intermixing, magnetic topological insulators, magnetic transitions

## Abstract

Magnetic topological insulators (TIs) herald a wealth of applications in spin‐based technologies, relying on the novel quantum phenomena provided by their topological properties. Particularly promising is the (MnBi_2_Te_4_)(Bi_2_Te_3_)_*n*_ layered family of established intrinsic magnetic TIs that can flexibly realize various magnetic orders and topological states. High tunability of this material platform is enabled by manganese–pnictogen intermixing, whose amounts and distribution patterns are controlled by synthetic conditions. Here, nuclear magnetic resonance and muon spin spectroscopy, sensitive local probe techniques, are employed to scrutinize the impact of the intermixing on the magnetic properties of (MnBi_2_Te_4_)(Bi_2_Te_3_)_*n*_ and MnSb_2_Te_4_. The measurements not only confirm the opposite alignment between the Mn magnetic moments on native sites and antisites in the ground state of MnSb_2_Te_4_, but for the first time directly show the same alignment in (MnBi_2_Te_4_)(Bi_2_Te_3_)_*n*_ with *n* = 0, 1 and 2. Moreover, for all compounds, the static magnetic moment of the Mn antisite sublattice is found to disappear well below the intrinsic magnetic transition temperature, leaving a homogeneous magnetic structure undisturbed by the intermixing. The findings provide a microscopic understanding of the crucial role played by Mn–Bi intermixing in (MnBi_2_Te_4_)(Bi_2_Te_3_)_*n*_ and offer pathways to optimizing the magnetic gap in its surface states.

## Graphical abstract

Intrinsic magnetic topological insulators of the Mn(Bi,Sb)2Te4‐family promise unprecedented spin control if their magnetic structure is not spoiled by unavoidable Mn‐Bi/Sb antisite defects. Nuclear magnetic resonance and muon spin spectroscopy reveal an unexpected but promising way toward this control: at about half of the magnetic critical temperature the antisites decouple from the magnetic structure suppressing the harmful magnetic disorder effects.

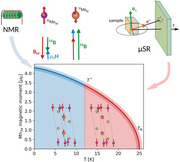

## 1 Introduction

The interplay between non‐trivial topology and magnetic order has been under the spotlight since the advent of a topological era in condensed matter physics because it may enable versatile and tunable topological phases.^[^ ^1^, ^2^, ^3^, ^4^, ^5^ ^]^ Magnetic topological materials emerged as an ideal platform for harbouring emergent quantum phenomena of technological relevance, including the quantum anomalous Hall effect (QAHE) and axion electrodynamics.^[^ ^6^, ^7^, ^8^, ^9^, ^10^ ^]^ A layered (van der Waals) compound MnBi_2_Te_4_, which is a magnetic derivative of the prototypical Bi_2_Te_3_ topological insulator, has been established as the first intrinsically magnetic TI.^[^ ^11^, ^12^, ^13^, ^14^ ^]^ In this compound, the local moments of the Mn atoms adopt an A‐type antiferromagnetic (AFM) order, consisting of a ferromagnetic (FM) alignment within the Mn layer, with AFM stacking in the perpendicular direction.^[^ ^11^, ^14^, ^15^ ^]^ Combination of a layered crystal structure and the A‐type AFM order makes MnBi_2_Te_4_  to fall under the *Z*_2_ topological classification of AFM insulators.^[^ ^16^ ^]^ The non‐trivial value of the invariant, *Z*_2_ = 1, stemming from the spin‐orbit coupling driven bulk band gap inversion, categorizes it as a 3D AFM TI.^[^ ^11^, ^12^, ^13^, ^14^ ^]^ In the 2D limit, thin MnBi_2_Te_4_  films were theoretically predicted^[^ ^17^, ^18^ ^]^ and experimentally confirmed to show the AFM axion insulator state,^[^ ^19^, ^20^ ^]^ as well as the quantized Hall effect, both under external field^[^ ^19^, ^20^, ^21^, ^22^ ^]^ and in remanence.^[^ ^22^ ^]^

On top of its exciting intrinsic properties, MnBi_2_Te_4_  also fosters a highly tunable material platform. Multiple tuning knobs, not only extrinsic such as magnetic field, pressure, and temperature, but also intrinsic such as Mn–Mn interlayer distance, variations of the chemical composition, and defect engineering, result in various magnetic and topological states. For example, various pnictogen or chalcogen substitutions and Mn/Bi/Te stoichiometry alternations give rise to such materials as MnSb_2_Te_4_, MnBi_2_Se_4_, or Mn_2_Bi_2_Te_5_, whose magnetic and topological properties have been studied both theoretically and experimentally.^[^ ^23^, ^24^, ^25^, ^26^, ^27^ ^]^ Furthermore, the van der Waals nature of MnBi_2_Te_4_  tolerates interlacing the adjacent (MnBi_2_Te_4_) septuple layers with various number *n* of nonmagnetic (Bi_2_Te_3_) quintuple layers, resulting in the (MnBi_2_Te_4_)(Bi_2_Te_3_)_*n*_ family of stacked structures (*n* = 1 for MnBi_4_Te_7_, *n* = 2 for MnBi_6_Te_10_, etc.).^[^ ^28^, ^29^ ^]^ The increasing distance between the septuple layers progressively weakens the interlayer exchange coupling with an increasing *n*, which enables an effective tuning of the magnetic structure by moderate magnetic fields,^[^ ^30^, ^31^, ^32^, ^33^, ^34^ ^]^ or hydrostatic pressure,^[^ ^35^ ^]^ driving these compounds from the AFM to the FM state. The (MnBi_2_Te_4_)(Bi_2_Te_3_)_*n*_ materials may host exotic, field‐induced topological phases^[^ ^30^, ^36^, ^37^ ^]^ and temperature‐dependent metamagnetic states.^[^ ^38^ ^]^

Native defects in these materials are lately in the center of attention thanks to their strong influence on the magnetic and electronic structure.^[^ ^39^, ^40^, ^41^, ^42^, ^43^, ^44^ ^]^ They are exploited as an effective tuning knob to purposely modify the latter.^[^ ^39^, ^45^, ^46^, ^47^ ^]^ These defects originate from antisite intermixing between the native manganese and pnictogen crystallographic sites. This phenomenon is favored by similar ionic radii, especially those of the Mn and Sb,^[^ ^48^ ^]^ which enables ca. three times stronger degree of intermixing in MnSb_2_Te_4_ compared to MnBi_2_Te_4_. Specifically, Mn atoms partially occupy the Sb/Bi 6*c* Wyckoff site (Mn_6*c*_ antisite), while pnictogen atoms swap to the Mn 3*a* site (Bi_3*a*_ antisite). The amounts of swapped cations do not necessarily fulfill the electroneutrality assumptions for Mn(II) and Sb(III)/Bi(III), and the occurrence of cationic vacancies in both sites is debated.^[^ ^49^ ^]^ Strong intermixing in MnSb_2_Te_4_  promotes the FM interlayer coupling.^[^ ^50^ ^]^ The magnetic transition temperature jumps from *T*_N_ = 19 K in the AFM‐like bulk Mn_1 − *x*_Sb_2 + *x*_Te_4_ single crystals^[^ ^39^ ^]^ to *T*_C_ = 58 K in the FM‐like Mn_1 + *x*_Sb_2 − *x*_Te_4_ ones,^[^ ^47^ ^]^ which is achieved by varying the growth conditions. (Hereinafter, the main magnetic transition is referred to as *T*_m_, meaning either *T*_N_ for AFM or *T*_C_ for FM samples.) Moreover, the interlayer coupling can become truly FM in MnBi_6_Te_10_ via Mn‐Bi defects engineering under appropriate growth conditions.^[^ ^45^, ^46^ ^]^ This FM coupling may not only help to realize an FM axion insulator state,^[^ ^36^, ^37^ ^]^ but also the QAHE in contrast to the AFM MnBi_2_Te_4_.^[^ ^22^, ^51^ ^]^

Low‐temperature neutron diffraction measurements, performed on both the AFM‐ and FM‐like MnSb_2_Te_4_ bulk single crystals, reveal that the local moments of the Mn_6*c*_ atoms are coupled antiparallel to the Mn_3*a*_ ones (*ferri*magnetic structure).^[^ ^39^, ^52^ ^]^ However, for the (MnBi_2_Te_4_)(Bi_2_Te_3_)_*n*_ family the magnetic role of the antisites has not been decisively established yet. Indeed, the available neutron diffraction data^[^ ^53^, ^54^, ^55^ ^]^ do not shed light on this issue (likely because of the much lower levels of intermixing as compared to MnSb_2_Te_4_), although the high‐field magnetization studies performed on MnBi_2_Te_4_ do suggest that there is an AFM coupling between the Mn_6*c*_ and Mn_3*a*_ sublattices.^[^ ^40^ ^]^

To close this important gap, this article systematically investigates the magnetic behavior of the antisites in polycrystalline (MnBi_2_Te_4_)(Bi_2_Te_3_)_*n*_ with *n* = 0, 1, 2 and in one MnSb_2_Te_4_  sample by means of local magnetic probes, namely nuclear magnetic resonance (NMR) (Section 2) and muon spin spectroscopy (µSR) (Section 3). Both techniques probe the dynamic and thermodynamic material properties, as well as the disorder introduced by the antisites. The measurements are performed in applied and in zero (ZF) external magnetic field, and ^55^Mn NMR provides direct local evidence of the nearly opposite relative spin alignment of Mn_3*a*_ and Mn_6*c*_ for all compounds studied here. Importantly, NMR reveals a magnetic order‐disorder transition in the Mn_6*c*_ sublattice of MnSb_2_Te_4_ well below *T*_m_ = 27 K. This clearly discriminates two regions in the MnSb_2_Te_4_ magnetic phase diagram: i) *T* < T* < *T*_m_, when the Mn_6*c*_ and Mn_3*a*_ moments are coupled antiferromagnetically; and ii) T* < *T* < *T*_m_, when the Mn_6*c*_ sublattice is paramagnetic‐like, whereas the Mn_3*a*_ one sustains its intra‐ and interlayer orders. µSR and bulk magnetometry measurements confirm the same order‐disorder transition in the Mn_6*c*_ sublattice of all studied (MnBi_2_Te_4_)(Bi_2_Te_3_)_*n*_ as well.

The discovery of the loss of the Mn_6*c*_ sublattice magnetic ordering is highly relevant in the context of the crucial role that Mn‐Bi intermixing plays in the reduction of the gap at the Dirac point in MnBi_2_Te_4_.^[^ ^41^, ^42^, ^43^, ^44^, ^56^, ^57^, ^58^, ^59^, ^60^, ^61^ ^]^

## 2 The Antisites From the NMR Perspective

In this and the next section, we describe a unified picture emerging from NMR and µSR, skipping the technical details of its derivation for the sake of clarity. The principles of the two techniques are briefly described in Section 5 and further important details are provided in the Supporting Information.^[^ ^62^ ^]^ Section 5 and the Supporting Information also give details about the sample preparation and characterization; the protocols are based on our previous works,^[^ ^28^, ^29^, ^30^, ^45^, ^48^, ^63^ ^]^ which ensures consistency between all our published series of samples in terms of structural and magnetic properties.

In ZF‐NMR, ^55^Mn nuclear spins (gyromagnetic ratio ^55^γ = 10.576 MHz/T) precess around a very large hyperfine field *B*_hf_, between 40 and 45 T at 1.4 K. *B*_hf_ is primarily due to the negative coupling, −A, to the on‐site moment *g*µ_*B*_ **S**, with much smaller positive couplings B to six nearest neighbors Mn_3*a*_, and even smaller distant dipole contributions, so that in first approximation, assuming parallel **S** in the layer, the NMR frequency is 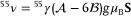.

### 2.1 NMR Peak Assignment

**Figure 1:**
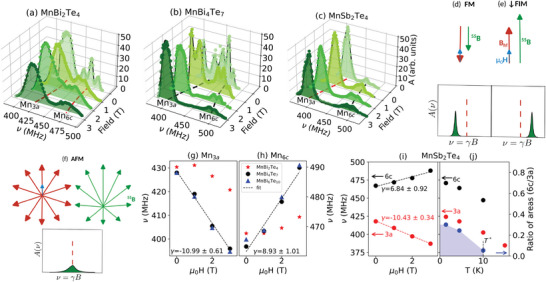
a–c), ^55^Mn NMR spectra of MnBi_2_Te_4_ (α sample), MnBi_4_Te_7_ and MnSb_2_Te_4_ respectively, at *T* = 1.4 K in increasing applied fields, starting from ZF; d–f) polycrystal vector composition ^55^ **B** = µ_0_ **H** + **B**_hf_ and their resulting spectral shifts for three simple cases: soft ferromagnet (FM, d), soft ferrimagnet, minority spin (↓FIM, e), antiferromagnet (AFM, f); g,h) field dependence of the Mn_3*a*_ and Mn_6*c*_ mean frequency peaks for the (MnBi_2_Te_4_)(Bi_2_Te_3_)_*n*_ family; i,j) Mn_3*a*_ and Mn_6*c*_ peaks for MnSb_2_Te_4_, field dependence (i) and temperature dependence (j) of their frequencies (black, red dots), temperature dependence of the ratio of their areas (blue dots, j).

A 3D view of the spectra for polycrystalline samples of MnBi_2_Te_4_, MnBi_4_Te_7_ and MnSb_2_Te_4_ at T = 1.4 K, in the frequency range 350 to 500 MHz, is shown in **Figure** **1**a–c (MnBi_6_Te_10_  in the Figure S8, Supporting Information^[^ ^62^ ^]^) with ZF at the back and increasing fields µ_0_ *H* towards the front. They all show two broad peaks patterns, each centered at a distinct frequency: ν_a, c_ = ^55^γ |^55^ **B**_a, c_|. Therefore two distinct Mn sites experience a different total local field modulus ^55^ *B*_a, c_, i.e., different values of *B*_hf_, as it would be expected for the main site Mn_3*a*_ and the anti‐site Mn_6*c*_. The 5–10% breadth of the frequency peaks is due to disorder in their vicinity, producing small variations of the local electronic environment, reflected in *B*_hf_.

The relative area under the two peaks at 3T assigns the lower‐frequency, majority peak to Mn_3*a*_ and the minority peak to Mn_6*c*_ (Figure 1a–c, the proportionality of the signal amplitude with the number of nuclei may not be guaranteed in ZF but it is recovered in 3T, see Section NMR). The frequencies confirm the assignment, since the six non‐negligible transferred couplings B of Mn_3*a*_ and the three nearly vanishing ones for Mn_6*c*_ are both opposite to the on‐site coupling A. This simple argument is confirmed by DFT simulations of the hyperfine coupling at the Mn sites (Figure S9, Supporting Information).

### 2.2 Antisite Spin Alignment

The same 3D plots (Figure 1a–c) show that the frequency splitting increases with the applied field. This can be understood from the field vector composition ^55^ **B** = µ_0_ **H** + **B**_hf_. In simple cases, the relative local orientation of the Mn_3*a*_ and Mn_6*c*_ magnetic moments is easily inferred from this vector composition, as shown in Figure 1d–f

(1)

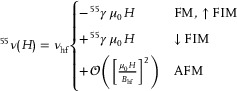




Namely, in the FM case, or equivalently, for the large magnetization sublattice of a ferrimagnet (↑ FIM) the moments align along the applied field, hence **B**_hf_ is opposite to **H** and the frequency decreases with increasing field. On the other hand, they anti‐align to **H** for the small magnetization sublattice of a ferrimagnet (↓ FIM), so there the frequency grows with **B**_hf_∥**H**. Finally, in the collinear AFM case, they do not align, and the superposition of all relative vector orientations in our polycrystalline samples leads to a broadening with no first order shift.^[^ ^62^ ^]^

Figure 1g,h shows the shifts vs. applied field for the (MnBi_2_Te_4_)(Bi_2_Te_3_)_*n*_ samples. The majority Mn_3*a*_ peak of MnBi_2_Te_4_ (red stars) does not shift with *H* up to 1 T, following the AFM behavior predicted by Equation (1). Its small shift for 2 T⩽*H* ⩽ 3 T is consistent^[^ ^62^ ^]^ with a canted antiferromagnetic (CAFM) state.^[^ ^64^, ^65^, ^66^ ^]^ In contrast, MnBi_4_Te_7_ and MnBi_6_Te_10_ (black and blue symbols, respectively) follow the FM case, like the MnSb_2_Te_4_ majority site, shown by the red symbols in Figure 1i, all fitted with negative slopes equal to γ = −^55^γ within error bars.

Neutron diffraction measurements on MnSb_2_Te_4_  have revealed the antiparallel alignment of the Mn_3*a*_ and Mn_6*c*_ local moments.^[^ ^39^, ^52^ ^]^ In our NMR data, this spin alignment is directly demonstrated in Figure 1i by the positive slope of ^55^ν(*H*) for the Mn_6*c*_ sites. Moreover, we see exactly the same behavior for all (MnBi_2_Te_4_)(Bi_2_Te_3_)_*n*_ materials (Figure 1g,h), i.e., their septuple layers show the same FIM structure as MnSb_2_Te_4_. While indications of this behavior in MnBi_2_Te_4_ were previously seen in high‐field magnetometry,^[^ ^40^ ^]^ the presented NMR results provide direct local evidence of the opposite spin alignment of Mn_3*a*_ and Mn_6*c*_ for *all* (MnBi_2_Te_4_)(Bi_2_Te_3_)_*n*_ with *n* = 0, 1 and 2. It is likely that the *n* > 2 members of the (MnBi_2_Te_4_)(Bi_2_Te_3_)_*n*_ family^[^ ^37^, ^67^, ^68^ ^]^ should also display this FIM structure, pretty much as their *n* = 0 − 2 analogs. Note that the ^55^ν(*H*) slope for (MnBi_2_Te_4_)(Bi_2_Te_3_)_*n*_ in Figure 1h is slightly reduced^[^ ^62^ ^]^ by an average canting angle θ between the sublattice magnetization and **H**, according to γ = ^55^γcos θ. This angle is small for the FM‐like materials, but quite sizable for CAFM MnBi_2_Te_4_, reflecting the large powder average angle between the magnetization of its canted Mn_3*a*_ sublattices and the field.

### 2.3 Antisite Moment Temperature Dependence

The ^55^Mn NMR signal quickly disappears when increasing the temperature above *T* = 1.4 K, as the NMR *T*_2_ relaxation time gets shorter than the instrumental dead‐time. For MnSb_2_Te_4_ the Mn_3*a*_ ZF NMR frequency (red symbols in Figure 1j) is detected up to *t* = *T*/*T*_m_ ≈ 0.6, whereas the Mn_6*c*_ ZF NMR frequency (black symbols) is detected only up to *t* = 0.4. They both decrease toward the second order transition at *T*_m_, following the order parameter. However, the ratio of their peak areas (blue symbol) decreases much more quickly over the same range, vanishing around *t* = 0.4. This implies that the Mn_6*c*_ peak disappears in a first‐order‐like fashion at a temperature T* well below *T*_m_.

Summarizing, NMR at 1.4 K directly detects the opposite alignment of the Mn_3*a*_ and Mn_6*c*_ sublattices in all of the systems studied here, and it demonstrates that in the Sb‐based compound the antisite Mn_6*c*_ disorders above T*, well below *T*_m_. The same information is not accessible for (MnBi_2_Te_4_)(Bi_2_Te_3_)_*n*_, due to a combination of lower ordering temperatures and shorter *T*_2_.

## 3 µSR Results

Spin‐polarized muons implanted in polycrystalline samples stop in few lowest energy interstitials. In ZF and for *T* < *T*_m_ the muon spin precesses around the local magnetic field *B*_µ_, due to ordered moments, producing coherent oscillations at a frequency γ_µ_ *B*_µ_ in the asymmetry of the muon decay at early times (γ_µ_ = 135.554 MHz/T, see Section 5).

**Figure 2:**
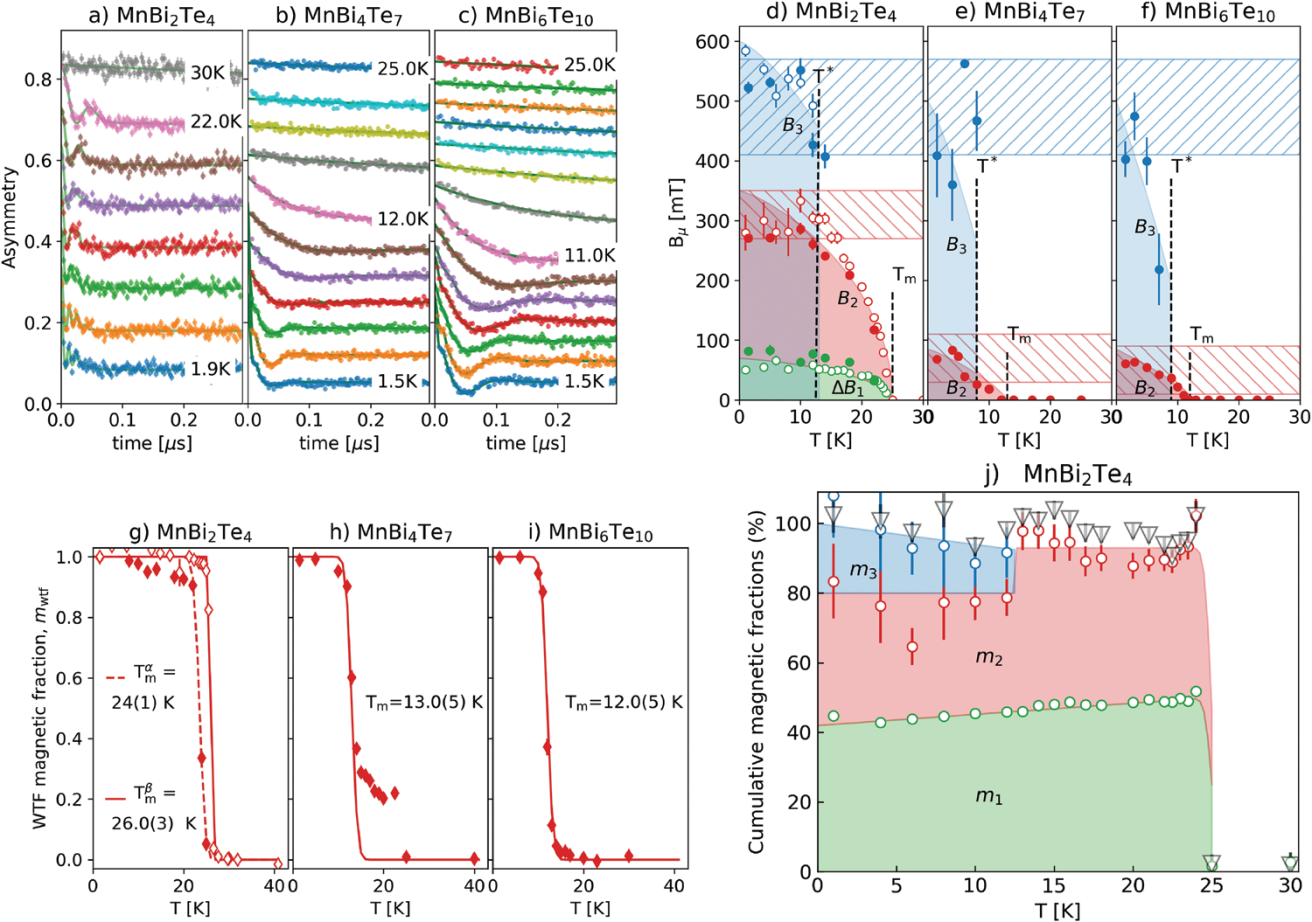
µSR in (MnBi_2_Te_4_)(Bi_2_Te_3_)_*n*_, *n* = 0, 1, 2. a–c) Early time ZF asymmetries at various temperatures with best fits (solid curve), displaced vertically for clarity; d–f) Temperature dependence of the internal fields Δ*B*_1_, *B*_2_, *B*_3_ (green, red, blue symbols, respectively), the hatched bands show the values predicted by DFT at *T* = 0, shaded areas are guides to the eye (Equation (7)); g–i) WTF magnetic volume fraction *m*_wtf_ versus temperature; j) ZF transverse cumulative magnetic fractions *m*_1_ (green circles), *m*_1_ + *m*_2_ (red circles) and *m*_1_ + *m*_2_ + *m*_3_ (blue circles), plus longitudinal magnetic fraction *m*_L_ (grey triangles), cfr. Equation (2). Data in panel (a) and open symbols in panel (d,g,j) correspond to the MnBi_2_Te_4_ β sample.

Selected ZF‐µSR early‐time asymmetries *A*(*t*) are shown in **Figure** **2**a–c for the (MnBi_2_Te_4_)(Bi_2_Te_3_)_*n*_ materials at different temperatures, with their best fit to a minimal model, Equation (2) in Section 5. The model describes the internal field distribution *p*(*B*_µ_) probed by muons as a few Gaussian components of very broad width Δ*B*_*i*_. Starting from MnBi_2_Te_4_ (Figure 2a), the asymmetry shows a fast, over damped initial relaxation and a second damped oscillation below *T*_m_. Both components correspond to appreciable internal fields, the fast initial Gaussian decay to a mean value smaller than its width 0 ≲ *B*_1_ < Δ*B*_1_, and the visible oscillation to an observable mean value *B*_2_ > Δ*B*_2_. At low temperature a third oscillating component (*B*_3_ > Δ*B*_3_) appears. By comparison, the *n* = 1, 2 members differ from *n* = 0 in *i)* the absence of the *B*_1_ < Δ*B*_1_ fast initial decay and *ii)* a lower field *B*_2_ value, whereas, they also display *iii)* a high field *B*_3_ component, that sets in only at lower temperatures. The presence of two distinct oscillations, both heavily damped, is more evident in the low temperature best fits of Figure S10 (Supporting Information).^[^ ^62^ ^]^

### 3.1 Magnetic Transitions

The temperature dependence of the internal fields, Δ*B*_1_(*T*) (the width of that distribution), *B*_2_(*T*) and *B*_3_(*T*), are shown in Figure 2d–f. It reveals two common features among all three family members: Δ*B*_1_ and *B*_2_ correspond to the order parameter and vanish at the second‐order magnetic transitions, *T*_m_; in contrast, *B*_3_ vanishes abruptly at T*, inside the ordered phase, without any corresponding anomaly in Δ*B*_1_, *B*_2_.

Weak transverse field (WTF) µSR provides the amplitude of the spin precession in a small applied field (µ_0_ *H* ≪ Δ*B*_1_, *B*_2_, *B*_3_). Below *T*_m_ this amplitude drops abruptly. The (MnBi_2_Te_4_)(Bi_2_Te_3_)_*n*_ *magnetic volume fraction* *m*_wtf_ shown in Figure 2g–i is obtained from WTF measurements (Equation (4) in Section 5) and demonstrates that MnBi_2_Te_4_ and MnBi_6_Te_10_ undergo very sharp transitions, at *T*_m_, (the width of the transition for a 90%–10% volume reduction is Δ*T* < 1 K), despite their relatively large atomic disorder implied by the presence of antisites. The *n* = 1 sample displays a sharp transition as well, but a 20% contribution, which we attribute to intergrowths of the MnBi_2_Te_4_ phase, is also visible (Figure 2h).

Similar results were obtained for MnSb_2_Te_4_.^[^ ^69^ ^]^ Notably, we measured two distinct MnBi_2_Te_4_ samples, labeled α and β (filled and open symbols, respectively, in Figure 2d,g,j), which readily show distinguishable transitions (*T*_m_ = 26.0(3), 24(1) K), due to slightly different preparation conditions. The residual WTF amplitude well below *T*_m_  is due to a small fraction of muons implanted outside the sample, that do not experience its spontaneous internal magnetic field distribution.

Separate magnetic volume fractions *m*_*i*_ are also derived from the ZF normalized amplitudes at each internal field *B*_*i*_ (Equation (5), Section µSR). Independently, the total volume fraction *m*_L_ is obtained from the longitudinal amplitude (Equation (6)). Figure 2j shows the cumulative sum of the three transverse fractions for MnBi_2_Te_4_‐β. Both *m*_L_ (grey symbols) and *m*_1_ + *m*_2_ (red symbols) drop sharply at the transition T_m_, in agreement with *m*_wtf_. In contrast, *m*_3_ disappears abruptly at T* = 12(1) K, suggesting that this component originates from muon sites sensitive to the subtle change that takes place across that point. This is reminiscent of our NMR MnSb_2_Te_4_ results. For the (MnBi_2_Te_4_)(Bi_2_Te_3_)_*n*_ samples, the temperature coincides with a clear anomaly appearing in magnetization (Figure S7 and Table SI, Supporting Information), suggesting that Mn_6*c*_ moment are undergoing fast (paramagnetic‐like) reorientations above T*. Further insight crucially requires the correct identification of the muon‐stopping sites.

### 3.2 The Antisites from the µSR Perspective

**Figure 3:**
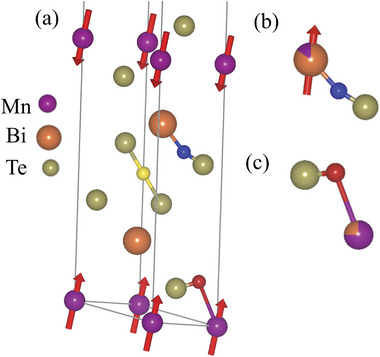
Representative muon sites as small colored atoms in MnBi_2_Te_4_ (and MnSb_2_Te_4_) by DFT+µ: Te‐µ‐Mn (red), Te‐µ‐Bi (blue atom), Te‐µ‐Te (yellow). a) Half the primitive cell with Mn_3*a*_ only, b) Te‐µ‐Mn@Bi  and c) Te‐µ‐Bi@Mn.

The muon sites were identified and their respective *T* = 0 K field values, *B*_µ_, were computed using a standard protocol, known as DFT+µ,.^[^ ^70^, ^71^, ^72^, ^73^ ^]^ These are explained briefly in Section 5 and discussed in more details in the Supporting Information.^[^ ^62^ ^]^ In the ideal MnBi_2_Te_4_ crystal, this protocol identifies three stable sites, shown in **Figure** **3** as Te‐µ‐Mn (red sphere), Te‐µ‐Bi (blue sphere), and Te‐µ‐Te (yellow sphere) and reported in the top box of **Table** **1**. The mean field values at these sites agree nicely with the experimental component *B*_1_ (Te‐µ‐Bi and Te‐µ‐Te) and *B*_2_ (Te‐µ‐Mn), respectively. Notice that the correspondence between sites and fields is not bijective (more sites may contribute to the same field distribution) and that the uncertainty in the DFT+µ derived values is below 25%.

**Table 1:** *T* = 0 local field at the representative muon sites in MnBi_2_Te_4_, identified by color as in Figure 3 (see Supporting Information for details.^[^ ^62^ ^]^) Filled circles refer to muon sites: first three rows, far from antisites; last two rows, nn to an antisite. Colored dashes refer to colors of symbols in Figure 2d.

Muon site label	nn antisite	DFT+µ *B*_µ_(mT)	Experiment	
			*B*_*i*_ (mT)	term
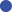 Te‐µ‐Bi	–	93	0(80)	– Δ*B*_1_
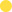 Te‐µ‐Te	–	0		
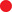 Te‐µ‐Mn	–	314	290(10)	– *B*_2_
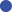 Te‐µ‐Mn@Bi	Mn_6*c*_	527	550(30)	– *B*_3_
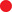 Te‐µ‐Bi@Mn	Bi_3*a*_	95	0(80)	– Δ*B*_1_

The presence of intermixing modifies these findings in two ways: i) inherent disorder broadens considerably all field distributions, producing large widths Δ*B*_*i*_, and ii) the extra moment modifies significantly the mean field values at muon sites nearest neighbor (nn) to Mn_6*c*_ and Bi_3*a*_. We label these two modified sites as Te‐µ‐Mn@Bi and Te‐µ‐Bi@Mn, respectively, and report their properties in the lower box of Table 1. Both these consequences are observed experimentally, in particular the calculation for the muon site Te‐µ‐Mn@Bi agrees with local field *B*_3_, while Te‐µ‐Bi@Mn contributes to *B*_1_.

Let us now turn to the (MnBi_2_Te_4_)(Bi_2_Te_3_)_*n*_ compounds with *n* = 1, 2, where the sites closest to the Mn_3*a*_ layer are predicted to be very similar to those of *n* = 0. The farther high symmetry Te‐µ‐Te site, instead, is replaced by more than one site in the intervening quintuple layers. Since all of them are far removed from Mn_3*a*_, we expect a large majority of muon sites characterized by very small or vanishing local field values. However, *n* = 1, 2 samples show non‐vanishing net magnetic moment, hence *B*_µ_ has an additional Lorentz field term *B*_L_ = 4π*M*/3 ≈ 70, 50 mT, respectively. This contributes negligibly to the high field of the Te‐µ‐Mn@Bi site, *B*_3_ ≫ *B*_L_, but significantly to the low field Te‐µ‐Bi@Mn, Te‐µ‐Bi and Te‐µ‐Te sites. This justifies both the second experimental field value *B*_2_ ≈ *B*_L_ (Figure 2e,f), its large fraction *f*_2_ and the disappearance of the *f*_1_, *B*_1_ = 0 signal.

The agreement of all these predictions, shown as hatched color bands in Figure 2d–f, with *T* → 0 K experimental data is altogether remarkable. It is the more so, in as much the same three DFT muon sites support a coherent, simple interpretation of the data for up to three experimental fit components, in four different compounds, over two magnetic phases.

## 4 Discussion and Conclusion

An important result from NMR is that the size of the moment on Mn obtained from the hyperfine field, in first approximation, is proportional to the on‐site coupling A, which is roughly 10 T/µ_*B*_ for all 3d ions and for ^55^Mn in particular.^[^ ^74^, ^75^, ^76^ ^]^ An estimate of this coupling comes from the ZF NMR frequency of Mn_3*a*_ and Mn_6*c*_. Taking the value of the latter for its much smaller transferred terms B, and, conservatively, half their difference as the uncertainty, we get roughly the same moment for all three (MnBi_2_Te_4_)(Bi_2_Te_3_)_*n*_ samples, µ_Mn_ = 4.3(2) µ_*B*_, in agreement with neutron diffraction.^[^ ^77^ ^]^

Importantly, fast Mn_6*c*_ spin fluctuations above T*  justify the disappearance of the *f*_3_ component, assigned consistently to the Te‐µ‐Mn@Bi site and mostly due to the nn Mn_6*c*_ moment. This observation is common to all three compositions. We recall that the same conclusion is drawn from the NMR findings on MnSb_2_Te_4_ (Section 2), confirmed by µSR as well.^[^ ^62^ ^]^ In addition, µSR results confirm the first order character of T*, since the internal field *B*_3_, proportional to the Mn_6*c*_ moment, is still large when the signal amplitude vanishes (Figure 2d–f, mimicking the NMR results of Figure 1j).

The anomaly at T* is confirmed by magnetization data,^[^ ^62^ ^]^ which, however, are obtained in an applied field. Recall that in MnBi_6_Te_10_ the local field *B*_2_ is assigned to the low field sites Te‐µ‐Bi, Te‐µ‐Te, and Te‐µ‐Bi@Mn,^[^ ^62^ ^]^ in the presence of an additional comparable Lorentz field, *B*_L_, due to the net domain magnetization. The smooth behavior of *B*_2_ across T* indicates that *B*_L_ survives also in zero applied field above the transition, suggesting that a dominant FM stacking of Mn_3*a*_ layers persists above T*  in zero field, therefore it is not induced by the external field.

The orientation of the antisite static moments successfully shows the sign of their dominant local exchange, displaying a universal behavior in this family, but this does not tell us the full magnetic structure of each material, that varies in the family. Actually, for MnBi_2_Te_4_  we can assume that *T*_m_ is a Néel transition and the sample is AFM in zero field. Here, T* (detected in ZF) clearly merges with the low temperature transition seen in *M*(*T*) (Figure S1, Supporting Information^[^ ^62^ ^]^). For MnBi_4_Te_7_ the presence of a cusp in *M*(*T*) suggests AFM bulk behavior at *T*_m_, but µSR shows the presence of an *n* = 0 contribution and we avoid further considerations. Finally, MnBi_6_Te_10_  was already shown to be FM under certain growth conditions,^[^ ^29^, ^45^, ^46^ ^]^ which we reproduce in this work as well.

**Figure 4:**
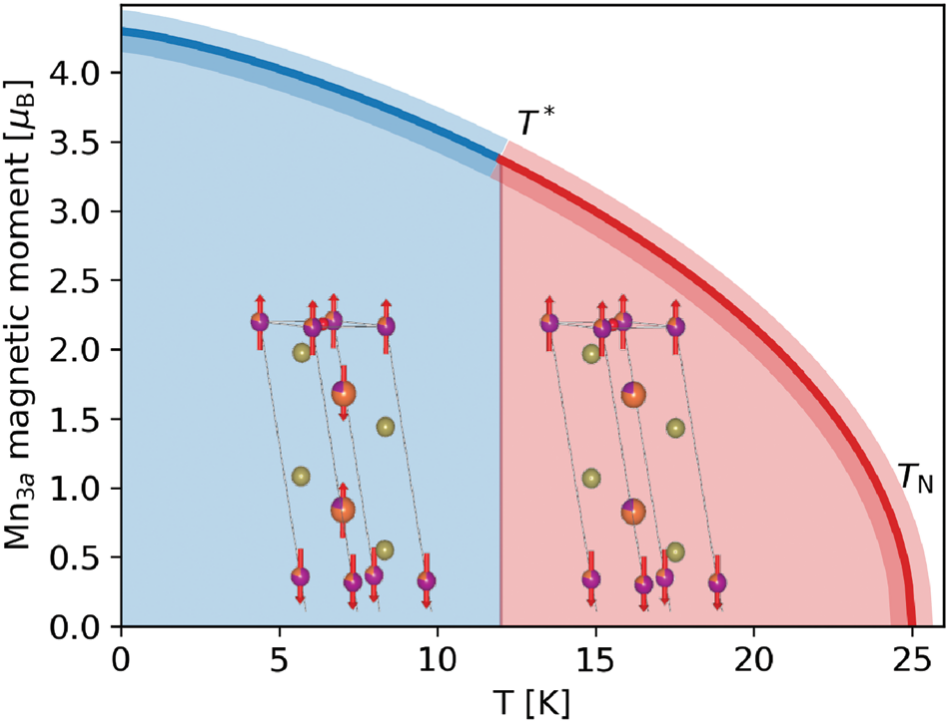
MnBi_2_Te_4_ phase diagram summary: temperature dependence of the Mn_3*a*_ magnetic moment, rescaled from the µSR fields Δ*B*_1_(*T*), *B*_2_(*T*), and *T* → 0 value obtained from ZF Mn_3*a*_ NMR (shaded bands translate uncertainty in the latter). Insets: magnetic structures in one septuple layer of the primitive cell.

**Figure** **4** summarizes our findings in a schematic phase diagram for the A‐type antiferromagnet MnBi_2_Te_4_, where the *T* → 0 magnitude of the static Mn moment is taken from NMR and its temperature behavior from the interpolation by Equation (7) on the ZF µSR fields Δ*B*_1_(*T*), *B*_2_(*T*), of Figure 2d. The blue region is characterized by the static ordering of the diluted Mn_6*c*_ moments, aligned antiparallel to the main Mn_3*a*_ moments, as NMR demonstrates. The static moment at Mn_6*c*_ vanishes in the red region.

A similar plot for MnBi_6_Te_10_ is reported in the Supporting Information^[^ ^62^ ^]^ (MnBi_4_Te_7_ data are less reliable due to the presence of a sizable fraction of MnBi_2_Te_4_ layer intergrowths, Figure 2h; Figure S4, Supporting Information). Remarkably, in all three compositions, the mean Mn_3*a*_ order does not change appreciably across T*.

The red‐shaded high‐temperature phase is characterized by a very sharp second‐order transition, as it is witnessed in all samples by the abrupt vanishing of the magnetic volume fraction, both by WTF and ZF µSR (Figure 2g–i). We highlight the unique ability of µSR, as opposed to both magnetometry and neutron scattering, to distinguish the reduction of the magnetic moment, encoded in the local field, from the volume fraction, encoded in the amplitude of the signal. Local disorder, like that expected from magnetically coupled, random antisites, could yield a distribution of transition temperatures, and hence a more progressive reduction of the volume fraction than that displayed in all our samples. This suggests that, from a magnetic point of view, the high‐temperature ordered phase approaches closely the ideal, intermixing‐free material. This provides both the antiferromagnetic and the ferromagnetic versions of a close‐to‐ideal magnetic topological insulator. This discovery is highly relevant in the context of the crucial role that Mn‐Bi intermixing plays in the reduction of the gap at the Dirac point in MnBi_2_Te_4_.^[^ ^41^, ^42^, ^43^, ^44^, ^56^, ^57^, ^58^, ^59^, ^60^, ^61^ ^]^ Indeed, a recent scanning tunneling spectroscopy study reveals that the surface gap is absent in the regions with a high degree of intermixing, while the areas with the lower degree of intermixing show gapped spectra.^[^ ^42^ ^]^ According to density functional theory calculations^[^ ^41^ ^]^ the antiferromagnetic coupling between the Mn_3*a*_ and Mn_6*c*_ sublattices is responsible for the gap reduction or suppression. Photoemission data^[^ ^41^, ^61^ ^]^ also show a gap reduction due to the presence of antisites; unfortunately they do not elucidate the gap behavior across T*, which remains an open question for further experiments.

Early sublattice decoupling is well documented, for instance in intermetallic compounds,^[^ ^78^, ^79^, ^80^, ^81^, ^82^, ^83^, ^84^, ^85^ ^]^ and it often involves exchange coupling frustration, which may also play a role in the present case.^[^ ^86^ ^]^ Since intermixing is a common feature for all materials, including cation species with similar radii, we speculate that early antisite disordering may take place more often than one thinks. Therefore, our findings may have a more general impact than just on the present materials.

## 5 Experimental Section

### Synthesis

Polycrystalline samples of (MnBi_2_Te_4_)(Bi_2_Te_3_)_*n*_ with *n* = 0, 1, 2 and of MnSb_2_Te_4_ were prepared by high‐temperature solid‐state reactions following in general the protocols described in the previous works.^[^ ^30^, ^45^, ^48^, ^63^ ^]^ The polycrystals were kept as non‐compacted powders for NMR experiments, and pressed into pellets for the µSR experiments. Single crystal experiments were also performed with both techniques with very similar results, although with much smaller signal to noise ratio, and the extensive studies were restricted to the polycrystalline samples.

Two MnBi_2_Te_4_  (denoted α and β) powdered samples were obtained by two distinct high‐temperature annealing routes. The MnBi_2_Te_4_  α‐sample was prepared from a mixture of pre‐synthesized MnTe and Bi_2_Te_3_ taken in the ratio 0.87:1.05. The powders were handled in an argon‐filled dry glovebox (MBraun), homogenized in a dry ball‐mill (Retsch, MM400) at 20 Hz for 20 min and then pressed into a 6‐mm pellet (2 tons, 30 s). The pressling was placed into a quartz ampoule, sealed off under dynamic vacuum (3 × 10^−3^ mbar) and annealed in a temperature‐controlled tube furnace (Reetz GmbH) following the procedure developed in ref. [63].

The polycrystalline MnBi_2_Te_4_  β‐sample was synthesized by co‐melting of pre‐synthesized MnTe and Bi_2_Te_3_ in an evacuated quartz ampoule at a temperature of about 980 °C for 12 h, followed by slow cooling down to 580 °C at the rate of 5° h^−1^. This temperature was kept for 12 h followed by air‐quenching. Then, the polycrystalline sample was ground as a fine powder and converted into a pellet and was then sealed in a quartz container under the pressure of 10^−4^ Pa. The ampoule was further heated up to 585 °C for 8 h, then kept at this temperature for about 240 h followed by air‐quenching. The grinding and annealing process was repeated twice to achieve a homogenized phase‐pure compound.

The MnBi_4_Te_7_ and MnBi_6_Te_10_ samples were obtained from a corresponding, stoichiometric mixture of the powdered binaries that were handled similar to the ones above. The former was annealed at 585 °C for 10 days (heating rate 1° h^−1^) and the latter was annealed at 575 °C for 4 days (heating rate 90° h^−1^). Both samples were water‐quenched.

The MnSb_2_Te_4_ sample was synthesized from a stoichiometric mixture of the elements (Sigma–Aldrich, 9N5, Mn reduced prior to synthesis) that was ball‐milled at 20 Hz for 20 min, pelletized and annealed at 550 °C for 8 days, and finally quenched.

### X‐Ray Diffraction and Energy‐Dispersive X‐Ray Spectroscopy

Phase purity of all samples but β was analyzed by powder X‐ray diffraction (PXRD) on a Malvern Panalytical Empyrean three diffractometer, employing Cu*K*α_1_ radiation (λ = 1.54059 Å) and set in Bragg‐Brentano geometry. Lattice parameters refinement was conducted by Le Bail method to ensure the correct assignment of the (MnBi_2_Te_4_)(Bi_2_Te_3_)_*n*_  phases. A PXRD pattern of the β‐sample was taken on a Bruker D2 PHASER diffractometer using Cu*K*α_1_ radiation within the scanning range of 2θ = 5 − 75. The details of phase analysis for all (MnBi_2_Te_4_)(Bi_2_Te_3_)_*n*_,samples are given in the Supporting Information (Figures S1–S6, Supporting Information); the obtained results were in line with the previously published results.^[^ ^28^, ^29^, ^30^, ^45^, ^63^ ^]^

MnSb_2_Te_4_ accommodates strong Mn/Sb intermixing^[^ ^47^, ^48^, ^87^ ^]^ and, therefore, adopt various total compositions. To achieve the highest accuracy in the determination of the chemical composition of the powdered sample, calibrated EDX measurements were conducted. For that, parts of the sample were cast into synthetic resin (versosit) pucks, sputtered with a gold layer and painted with conductive silver paint to avoid charge accumulation. EDX spectra were recorded with a high‐resolution SEM EVOMA 15 (Zeiss) equipped with a Peltier‐cooled Si(Li) detector (Oxford Instruments) employing 30 kV acceleration voltage. Element quantification was obtained from least‐square fitting of edge models (Mn‐K, Te‐L, Sb‐L) invoking k‐factor calibration from the stoichiometric samples of similar composition (Sb_2_Te_3_ and MnTe). To assess systematic errors stemming from the different edge and reference choices, Sb_2_Te_3_ and MnTe references are included for Te in the quantification statistics. The determined composition of the sample was Mn_0.87(1)_Sb_2.03(1)_Te_4.00(1)_, which was in line with its magnetic properties reported in Ref. [69].

### NMR

The NMR spectra were measured in a He‐flow cryostat by means of the HyReSpect home‐built phase coherent broadband spectrometer.^[^ ^88^ ^]^ Spin‐echoes were excited at discrete frequency points by refocusing P–τ–P Hahn radio‐frequency (rf) pulse sequences, with optimized pulse duration and intensity to maximize the resonance signal, and shortest τ delay (limited by the apparatus dead time of few µs).

Spectra were reconstructed from the maximum of the spin‐echo Fourier transform amplitude at each frequency, corrected for the frequency‐dependent sensitivity and nuclear Boltzmann factors. The normalized values correspond to the spectral distribution of hyperfine fields at the ^55^Mn nuclei. When the best fit of the two spectra (ζ = Mn_3*a*_, Mn_6*c*_) require more than one Gaussian component each, their mean frequency was calculated from the corresponding weights *A*_α, *i*_ as the first moment ∑_*i*_ *A*_ζ*i*_ν_ζ*i*_/∑_*k*_ *A*_ζ.*k*_.

ZF nuclear echoes were collected with a non‐resonant probe circuit, by virtue of the large rf enhancement $\eta =H_{1}^{*}/H_{1}$, where *H*_1_ was the applied field at the radio frequency ω, and $H_{1}^{*}$ is the ω oscillating component of the huge hyperfine field *B*_hf_, following the electronic moment response to *H*_1_.^[^ ^89^, ^90^ ^]^ The factor η was very sensitive to nanoscopic and mesoscopic changes in the nucleus environment and this did not guarantee a uniform proportionality between spectral area and number of resonating nuclei. Relative proportionality was recovered in the more uniform resonant conditions obtained at high static applied fields.

### µSR

The µSR experiments were carried out at the Paul Scherrer Institute, Villigen, Switzerland, on the GPS spectrometer.

A minimal choice for the best‐fit function of the time domain ZF asymmetry, arising from parity violation in the weak muon decay, was the following

(2)

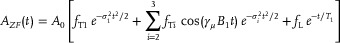

distinguishing the fast relaxing, precessing fractions $f_{T}=\sum_{i=1}^{3}f_{Ti}$ (T for transverse with respect to the initial muon spin direction) from the slow relaxing fraction *f*_L_ (L for longitudinal), which corresponds to local field components parallel to the initial muon spin direction. The precession relaxation rates were due to the width of each field distribution, σ_*i*_ = 2πγ_µ_Δ*B*_*i*_, their fractions obey *f*_T_ + *f*_L_ = 1 and the maximum experimental asymmetry, *A*_0_, is calibrated at high temperature. Polycrystalline averaging leads to 2*f*_L_ = *f*_T_, but a very fast transverse decay and a small fraction of muons stopping outside the sample may relax this ideal condition.

The WFT time domain signals were best fitted by an oscillatory and two relaxing functions

(3) $$\begin{array}{ccc} A_{TF}\left(t\right) & = & A_{0}\left[f_{p}\;\cos\left(\gamma B+\phi \right)\;e^{-\lambda_{p}t}+f_{T}\;e^{\sigma_{T}^{2}t^{2}/2}+f_{L}\;e^{-\lambda_{L}t}\right] \end{array}$$

where ϕ is an initial phase and *f*_*p*_ is the muon fraction that does not experience strong local hyperfine fields, which includes muons stopping outside the sample and inside paramagnetic microdomains. The former corresponds to the low temperature residual value *f*_*p*0_, whereas for *T* ≪ *T*_m_, one has *f*_*p*_ = 1, *f*_T_ = *f*_L_ = 0. The two relaxing function represent the transverse and longitudinal fractions of muons experiencing internal fields.

Three independent determinations of the magnetic volume fraction were given by

(4) $$\begin{array}{ccc} m_{\mathrm{wtf}}\left(t\right) & = & \frac{f_{p}\left(T\right)-f_{p0}}{1-f_{p0}} \end{array}$$

(5) $$\begin{array}{ccc} m_{T} & = & \sum_{i=1}^{3}m_{i}\;m_{i}=\frac{f_{Ti}}{f_{t0}} \end{array}$$

(6) $$\begin{array}{ccc} m_{L} & = & =\frac{3}{2}\left(1-f_{L}\left(T\right)\right) \end{array}$$

where *f*_T0_ = lim_*T* → 0_ *f*_T_(*T*) and Equation (5) neglects *f*_*p*0_, since in ZF *f*_L_ is indistinguishable from *f*_*p*_. They are used in Figure 2g,h (Equation (4)) and j (Equation (5) colored and gray symbols, for Equations (5) and (6), respectively).

Lastly, the internal fields Δ*B*_1_ = σ_1_/2πγ_µ_, and *B*_2_ proportional to the order parameter are fitted to a standard phenomenological function^[^ ^91^ ^]^

(7) $$b\left(T\right)={\left[1-{\left(\frac{T}{T_{m}}\right)}^{\gamma }\right]}^{\delta }$$

used both in the shaded colors of Figure 2d–f and in Figures 4 and Figure S8 (Supporting Information).

### DFT

The DFT calculation protocols for determining the muon implantation sites^[^ ^70^ ^]^ and the contact contribution to the hyperfine interactions in magnetic compounds^[^ ^71^, ^72^ ^]^ were well established. State of the art DFT calculations were performed on a magnetic supercell, including an extra impurity hydrogen atom. Sampling of the starting impurity supercell coordinates and lattice relaxation by force minimization yield minimum total energy sites and their spin couplings, that allow the calculation of the local field.^[^ ^73^ ^]^ Full calculation details are reported in the Supporting Information.^[^ ^62^ ^]^

## Conflict of Interest

The authors declare no conflict of interest.

## Supporting information

Supporting Information

## Data Availability

The original data are available at http://musruser.psi.ch, instrument GPS, years 2020, 2021, title includes the chemical formula. NMR data, the DFT input and results are available at Materials Cloud Archive https://archive.materialscloud.org/record/2024.20 DOI:10.24435/materialscloud:he-f5.
