# Supplementary material for: Ubiquitous Order‐Disorder Transition in the Mn Antisite Sublattice of the (MnBi2Te4)(Bi2Te3) n Magnetic Topological Insulators
Source: Adv Sci (Weinh). 2024 Jul 8;11(34):2402753. doi: 10.1002/advs.202402753 (PMC11425889; doi:10.1002/advs.202402753)
Supplement: Supplementary file 1 — Supporting Information [file ADVS-11-2402753-s001.pdf]

ADVANCED SCIENCE
Open Access

Supporting Information

for *Adv. Sci.*, DOI 10.1002/advs.202402753

Ubiquitous Order-Disorder Transition in the Mn Antisite Sublattice of the $(MnBi_2Te_4)(Bi_2Te_3)_n$ Magnetic Topological Insulators

Manaswini Sahoo, Ifeanyi John Onuorah, Laura Christina Folkers, Ekaterina Kochetkova, Evgueni V. Chulkov, Mikhail M. Otrokov, Ziya S. Aliev, Imamaddin R. Amiraslanov, Anja U. B. Wolter, Bernd Büchner, Laura Teresa Corredor, Chennan Wang, Zaher Salman, Anna Isaeva, Roberto De Renzi and Giuseppe Allodi*

Ubiquitous order-disorder transition in the Mn antisite sublattice of the $MnBi_2Te_4 \cdot n(Bi_2Te_3)$ magnetic topological insulators

M. Sahoo,[1, 2, 3, 4, *] I.J. Onuorah,[4, *] L.C. Folkers,[2, 3] E. Kochetkova,[5, 2] E.V. Chulkov,[6, 7, 8, 9] M.M. Otrokov,[10] Z.S. Aliev,[11, 12] I.R. Amiraslanov,[12] A.U.B. Wolter,[1] B. Büchner,[1, 2, 3] L. T. Corredor,[1] Chennan Wang,[13] Z. Salman,[13] A. Isaeva,[1, 5, 14, 15] Roberto De Renzi,[4, †] and G. Allodi[4]

[1]*Leibniz IFW Dresden, Helmholtzstraße 20, D-01069 Dresden, Germany*
[2]*Institut für Festkörper- und Materialphysik, Technische Universität Dresden, 01062 Dresden, Germany*
[3]*Würzburg-Dresden Cluster of Excellence ct.qmat, Germany*
[4]*Dipartimento di Scienze Matematiche, Fisiche e Informatiche, Universitá di Parma, Parco delle Scienze 7A, I-43124 Parma, Italy*
[5]*Van der Waals-Zeeman Institute, Department of Physics and Astronomy, University of Amsterdam, Science Park 094, 1098 XH Amsterdam, The Netherlands*
[6]*Donostia International Physics Center, 20018 Donostia-San Sebastián, Spain*
[7]*Departamento de Polímeros y Materiales Avanzados: Física, Química y Tecnología, Facultad de Ciencias Químicas, Universidad del País Vasco UPV/EHU, 20018 Donostia-San Sebastián, Spain*
[8]*Centro de Física de Materiales (CFM-MPC), Centro Mixto (CSIC-UPV/EHU), 20018 Donostia-San Sebastián, Spain*
[9]*Saint Petersburg State University, 199034 Saint Petersburg, Russia*
[10]*Instituto de Nanociencia y Materiales de Aragón (INMA), CSIC-Universidad de Zaragoza, 50009 Zaragoza, Spain*
[11]*Baku State University, AZ1148 Baku, Azerbaijan*
[12]*Institute of Physics Ministry of Science and Education Republic of Azerbaijan, AZ1143 Baku, Azerbaijan*
[13]*Laboratory for Muon Spin Spectroscopy, Paul-Scherrer-Institute, CH-5232 Villigen PSI, Switzerland.*
[14]*Faculty of Physics, Technical University of Dortmund, Otto-Hahn-Str. 4, 44221 Dortmund, Germany*
[15]*Research Center Future Energy Materials and Systems (RC FEMS), Germany*

(Dated: June 17, 2024)

I. SAMPLE PHASE ANALYSIS

Phase-purity of the obtained polycrystalline batches except for $MnBi_2Te_4$-β was evaluated by the Le Bail peak-profile refinement in the JANA2006 software package [1]. Results are presented in the Fig. S.1,S.2,S.3 and S.6 below, and confirm high crystallinity and overall homogeneity of the obtained products. In general, these results are strongly consistent with our previous works [2–5] where the Mn/Bi and Mn/Sb intermixing patterns were quantified by X-ray diffraction method. Fig. S.5 shows the powder X ray diffraction (PXRD) pattern of the phase-pure $MnBi_2Te_4$-β sample.

Small amounts of a non-magnetic Bi_2Te_3 impurity can be identified in the Fig. S.1 and Fig. S.3; its fraction varied from 0 to 10 wt. % amongst the studies samples prepared by the same method. The polycrystalline $MnBi_4Te_7$ appeared as phase-pure according to Fig. S.2. After the NMR and μSR measurements were performed, we realized, however, that some parts of this batch were less homogeneous and contained co-crystallized fractions of $MnBi_2Te_4$ (up to 20-25 wt. %) and $MnBi_4Te_7$ (see an exemplary PXRD pattern in Fig. S.4 and the discussion about Fig. 2 h in the main text). Occurrence of such local inhomogeneities is at all not surprising given that the differences between the solidification points of $MnBi_2Te_4$ and $MnBi_4Te_7$ is only 4°C according to the DSC results in [3].

The $MnSb_2Te_4$ sample is essentially phase-pure (Fig. S.6) with very low, unquantifiable imprurity of $MnTe_2$ (below 2 wt.%).

II. BULK MAGNETIC MEASUREMENTS

A Quantum Design Superconducting Quantum Interference Device Vibrating Sample Magnetometer (SQUID VSM) was utilized to conduct bulk DC magnetic measurements on the same samples employed for the NMR and μSR experiments. The temperature dependence of the magnetization is shown in Fig. S.7, for both zero-field cooled (ZFC) and field-cooled (FC) protocols, as measured over the temperature range 1.8 K to 60 K.

In the $MnBi_2Te_4$ sample, for $\mu_0 H=$ 10 mT, a cusp in $M(T)$ is observed at $T_N \simeq 24.6$K (see Fig. S.7a, in coincidence with a peak in dM/dT), indicating the onset of long-range antiferromagnetic order. Upon further cooling, both ZFC and FC curves exhibit an anomaly, appearing first as an upturn below 15 K, while below 11.5 K the ZFC and FC curves separate, denoting the onset of a ferromagnetic-like character at $T^* = 11.5$ K, as indicated by a dip in dM/dT. This second transition for $MnBi_2Te_4$ is suppressed in higher applied fields around 0.5 T, which shows the field-induced character of the transition.

A similar double transition is evidenced by the double

* MS and IJO contributed equally
† Corresponding address: roberto.derenzi@unipr.it

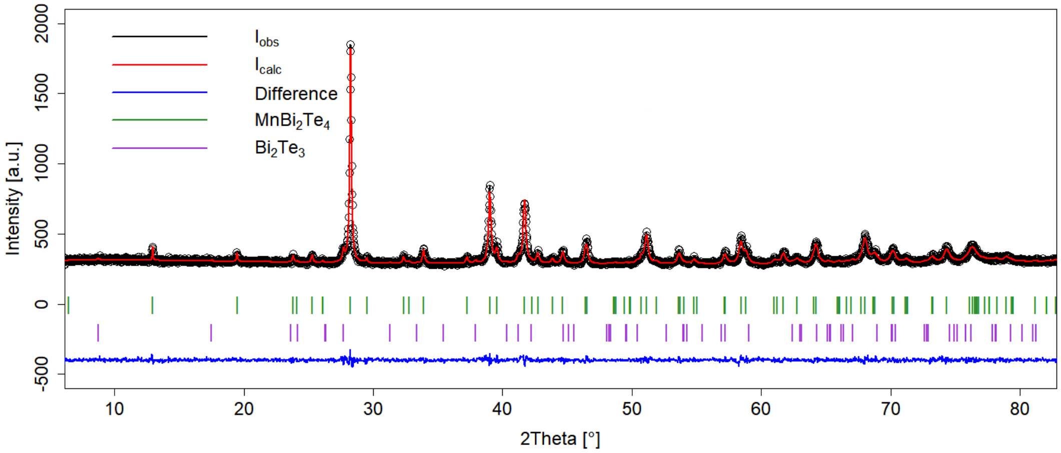

FIG. S.1: Le Bail analysis of the $MnBi_2Te_4$-α sample ($R_p = 0.10$, $R_{wp} = 0.13$, GoF $= 1.1$). The refined lattice parameters of $MnBi_2Te_4$ are (sp. gr. $R\bar{3}m$): $a = 4.3283(2)$ Å, $c = 40.978(2)$ Å. The refined lattice parameters of Bi_2Te_3 (5 wt. %) are (sp. gr. $R\bar{3}m$): $a = 4.3770(8)$ Å, $c = 30.400(8)$ Å.

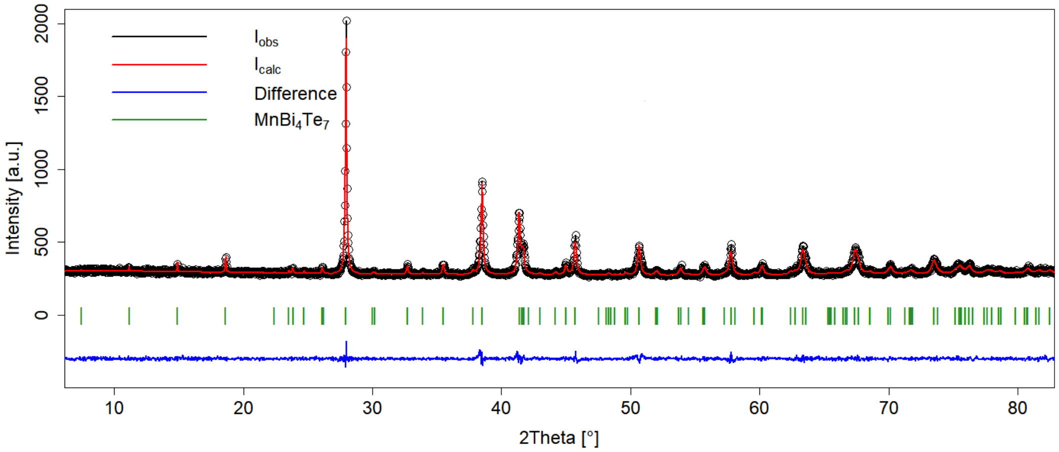

FIG. S.2: Le Bail analysis of the $MnBi_4Te_7$ sample ($R_p = 0.11$, $R_{wp} = 0.14$, GoF $= 1.1$). The refined lattice parameters are (sp. gr. $P\bar{3}m1$): $a = 4.3625(2)$ Å, $c = 23.794(1)$ Å.

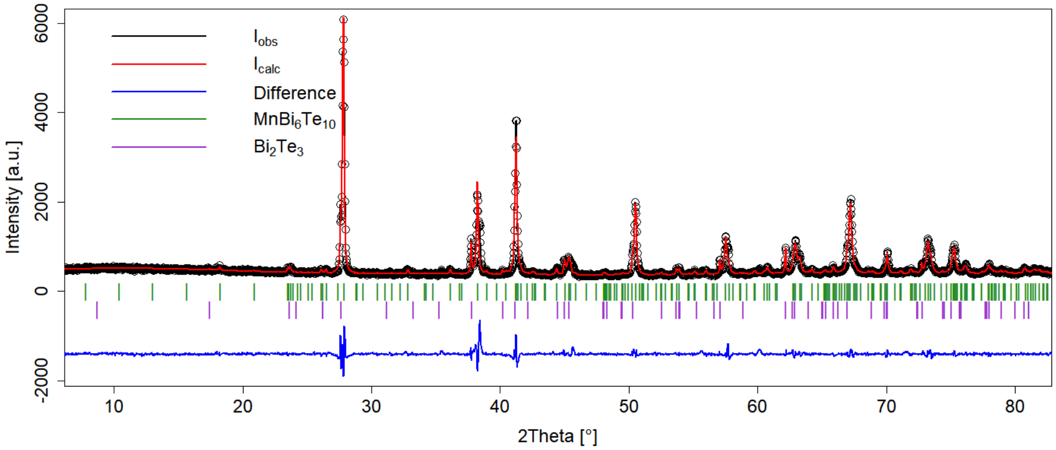

FIG. S.3: Le Bail analysis of the $MnBi_6Te_{10}$ sample ($R_p = 0.08$, $R_{wp} = 0.12$, GoF $= 1.8$). The refined lattice parameters of $MnBi_6Te_{10}$ are (sp. gr. $R\bar{3}m$): $a = 4.3710(1)$ Å, $c = 101.960(4)$ Å. The refined lattice parameters of the Bi_2Te_3 admixture (10 wt. %) are (sp. gr. $R\bar{3}m$): $a = 4.3826(2)$ Å, $c = 30.548(2)$ Å.

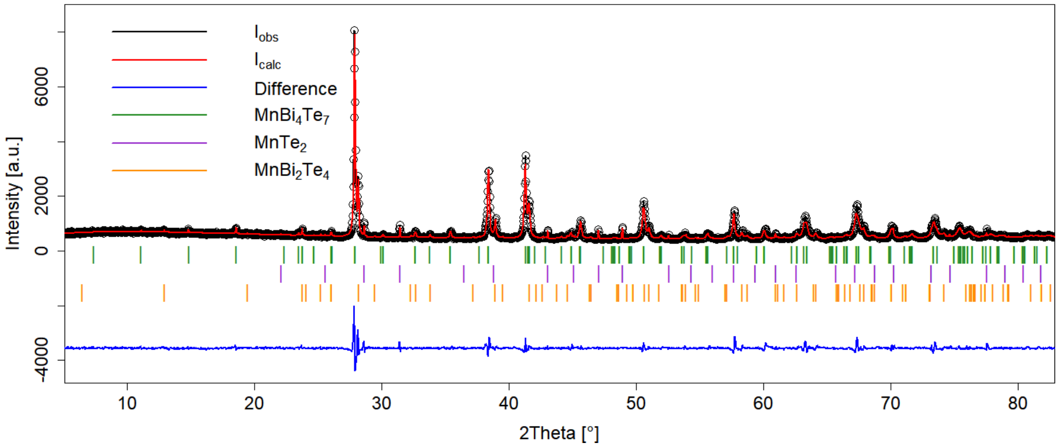

FIG. S.4: Le Bail analysis ($R_p = 0.08$, $R_{wp} = 0.10$, GoF = 1.9) of the sample with $MnBi_4Te_7$ (ca. 70 wt. %), $MnBi_2Te_4$ (ca. 23 wt. %) and $MnTe_2$ (ca. 7 wt. %). The refined lattice parameters are: $MnBi_4Te_7$ (sp. gr. $P\bar{3}m1$) $a = 4.3683(1)$ Å, $c = 23.852(1)$ Å; $MnBi_2Te_4$ (sp. gr. $R\bar{3}m$) $a = 4.3371(2)$ Å, $c = 41.021(4)$ Å; $MnTe_2$ (sp. gr. $Pa\bar{3}$) $a = 6.9588(2)$ Å.

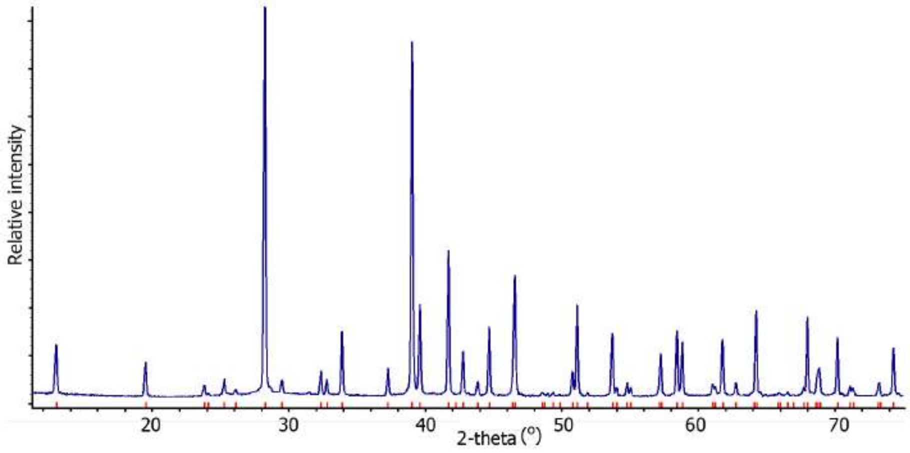

FIG. S.5: Experimental (blue) PXRD for $MnBi_2Te_4$-β. The red are the Bragg reflection positions for the phase.

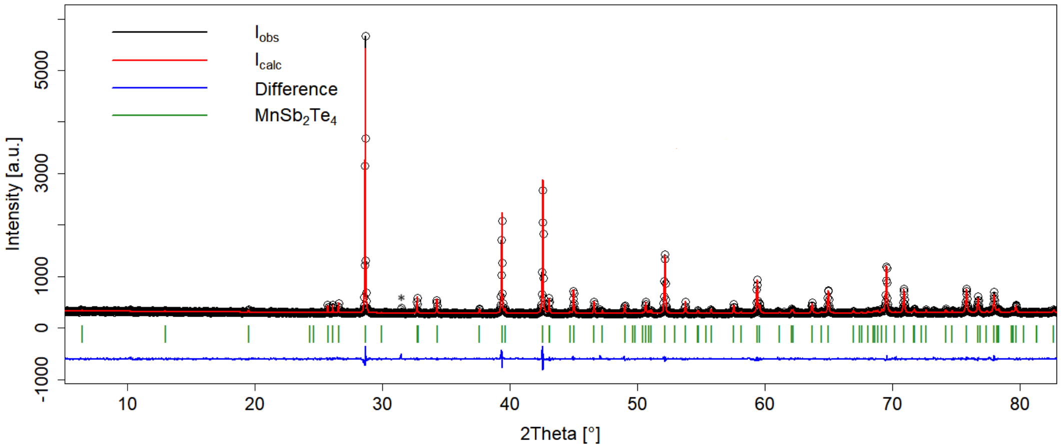

FIG. S.6: Le Bail analysis of the $MnSb_2Te_4$ sample ($R_p = 0.10$, $R_{wp} = 0.14$, GoF = 1.2). The highest-intensity peak of $MnTe_2$ is marked by a star symbol. The refined lattice parameters of $MnSb_2Te_4$ are (sp. gr. $R\bar{3}m$): $a = 4.24144(3)$ Å, $c = 40.8810(5)$ Å.

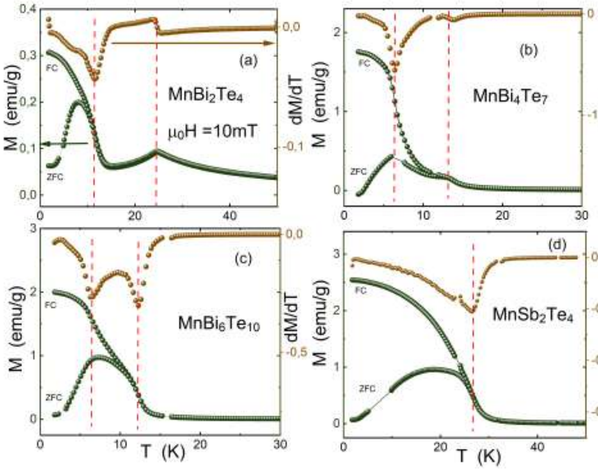

FIG. S.7: Temperature dependence of the magnetization $M(T)$ and of dM/dT in μ_0H= 10 mT, for (a) $MnBi_2Te_4$-α, (b) $MnBi_4Te_7$, (c) $MnBi_6Te_{10}$ and (d) $MnSb_2Te_4$.

dip in dM/dT observed in $MnBi_4Te_7$ and $MnBi_6Te_{10}$ as well (see Fig. S.7 b,c), although the AFM cusp is not directly detected in the latter sample. All the transition temperatures are listed in Tab. S.I. Notably, both transition temperatures observed in bulk magnetization are consistent with the corresponding features observed in μSR, indicating that the anomaly at T^* corresponds to a true thermodynamic phase transition.

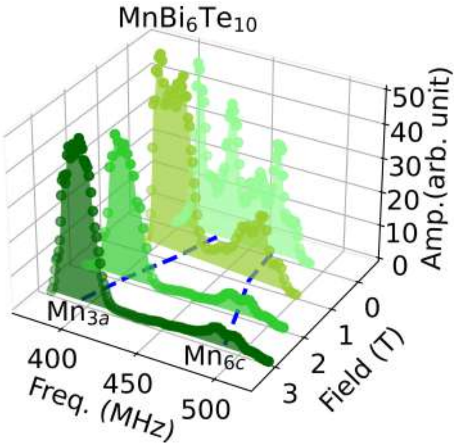

FIG. S.8: ^{55}Mn NMR spectrum of $MnBi_6Te_{10}$ at $T = 1.4$ K in increasing applied fields, starting from ZF. Blue dashed lines show the field dependence of the mean frequency for the Mn_{3a} and Mn_{6c} peaks.

In the $MnSb_2Te_4$ sample, a single ferromagnetic-like transition at T_C= 26.6K (see Fig. S.7 d) was identified, and the two-transition behavior observed in the $MnBi_2Te_4$ samples was absent. This absence is attributed to the dominant ferromagnetic signal stemming from Mn_{3a} in the bulk.

	T_m [K]		T^* [K]	
Sample	SQUID	μSR	SQUID	μSR
$MnBi_2Te_4$-α	24.6(1)	24(1)	11.5(1)	12(1)
$MnBi_4Te_7$	13.7(1)	13(1)	6.4(1)	6(1)
$MnBi_6Te_{10}$	12.3(1)	12(1)	6.5(1)	6(1)
$MnSb_2Te_4$	26.6(1)	-	-	-

TABLE S.I: Transition temperatures observed from both bulk magnetization and μSR.

III. NMR

A. Field dependence of ^{55}Mn NMR in $MnBi_2Te_4$

The dashed lines on the horizontal plane in Fig. 1 a, main paper, show that the mean frequencies of the NMR Mn_{3a} and Mn_{6c} peaks shift slightly with applied field. These frequencies are plotted as red stars in Fig. 1 g, h, for Mn_{3a} and Mn_{6c} respectively. Their slope can be appreciated by eye, an effective $\gamma = d\nu/dH\,/\mu_0$ that vanishes for $\mu_0H \leq 1$ T as predicted by Eq. 1 for the AFM case. Indeed, the hyperfine field in an AFM polycrystalline sample, depicted as red arrows in the cartoon (Fig. 1 f) adds in all possible crystal grain orientations to the external field (blue arrow), leading to different total local fields $B_{\rm hf}$ (green arrows), evenly distributed in modulus about the external field value. Recalling that $^{55}\nu = {}^{55}\gamma|{}^{55}\mathbf{B}|$ this leads to a vanishing shift in first order. The absolute value of γ increases around 2 T, as expected in a canted antiferromagnet (CAFM) polycrystal. Indeed, a crystal grain whose hexagonal c axis forms an angle θ with the field undergoes a spin flop transition at $2 \lesssim \mu_0H(\theta) \leq 3.57$ T. [6–8] The powder average results in a distribution of ^{55}B values (like in the pure AFM case), albeit with a mean shift, due to the coupling to the field ($\gamma < 0$ for $\uparrow Mn_{3a}$, $\gamma > 0$ for $\downarrow Mn_{6c}$).

For comparison, in the FIM case the spin alignment to the field is complete at any orientation and the corresponding Mn_{3a} (Fig. 1 g) and Mn_{6c} (Fig. 1 h) effective γ values for $MnBi_4Te_7$ and $MnBi_6Te_{10}$ (black and blue symbols, respectively) coincide with $\pm^{55}\gamma$ within errors, as expected for the $\uparrow$ and $\downarrow$ sublattice respectively.

B. ^{55}Mn NMR spectra of $MnBi_6Te_{10}$

A 3D view of the spectra for our polycrystalline sample of $MnBi_6Te_{10}$ in the frequency range 350 to 500 MHz, is shown in Fig. S.8, with ZF at the back and increasing fields μ_0H towards the front.

IV. DFT SIMULATION OF ^{55}Mn-NMR SPECTRA

We have calculated the hyperfine field at the Mn nucleus for both $MnBi_2Te_4$ and $MnSb_2Te_4$, first, without

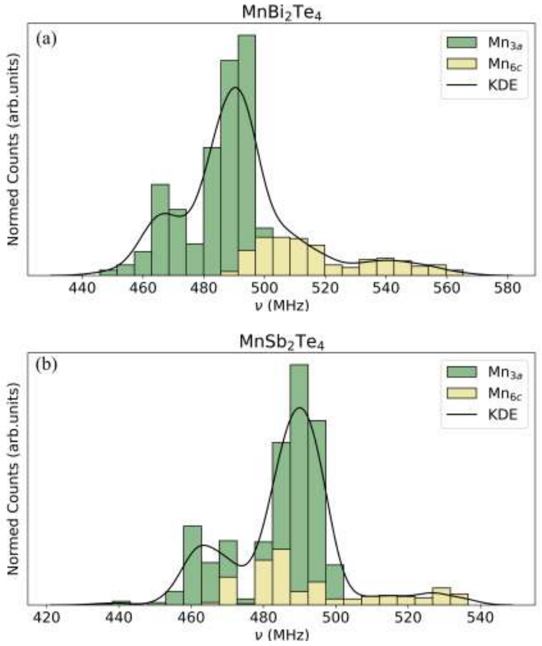

FIG. S.9: ^{55}Mn-hyperfine spectra calculated by DFT in $MnBi_2Te_4$ (a) and $MnSb_2Te_4$ (b), distinguishing the contributions from Mn_{3a} (green bars) and Mn_{6c} (yellow bars). In the calculation, the Mn_{6c} moment is assumed antiparallel to the moment of the nearest neighbor Mn_{3a} in both cases. The solid black line is the probability density distribution estimated by the Kernel density function (KDE).

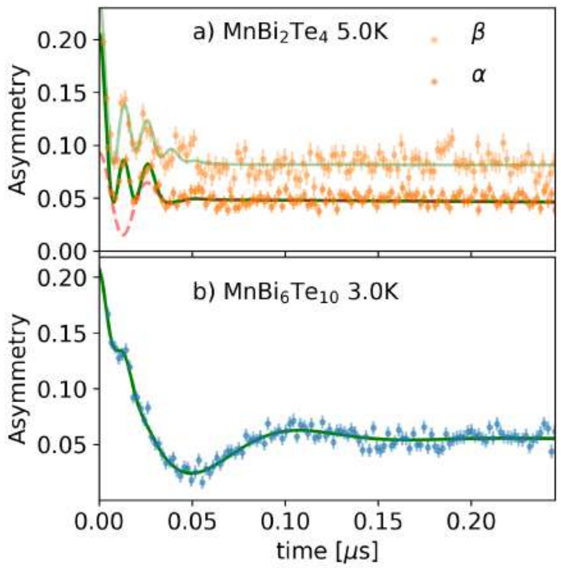

FIG. S.10: Best fits to Eq. 2, at low temperature for a) $MnBi_2Te_4$ α and β samples (dashed curve: best fit with components 1,3 set to zero amplitude to highlight component 2), and b) $MnBi_6Te_{10}$, with reduced $\chi^2 = 1.00, 0.98, 0.98$ respectively.

site intermixing, by an all electron DFT+U protocol as implemented in the ELK code [9]. We have used a dense 15×15×3 Monkhorst-Pack Mesh for $MnBi_2Te_4$ and 15×15×7 for $MnSb_2Te_4$. The muffin-tin radius of 2.4 a.u. for Mn, 2.8 a.u. for Bi, 2.6 a.u. for Sb and 2.6 a.u. for Te were used. The maximum length or cutoff for the $\mathbf{G}+\mathbf{K}$ vectors is 8.0 a.u., divided by the average of the muffin-tin radii. The hyperfine field at the Mn_{3a} nucleus in the ideal $MnBi_2Te_4$ and $MnSb_2Te_4$, calculated within the scalar relativistic approach, is 42.2 T and 45.6 T, corresponding to NMR frequencies of 446 MHz and 482 MHz, respectively. These values are in reasonable agreement with the experiments, being within 15% of the values from Fig. 1 g,i (430 and 423 MHz, respectively). For $MnSb_2Te_4$, the fully relativistic approach, better accounting for spin orbit coupling, yields a magnitude of almost coincident value of 45.5 T, showing that the dominant contribution is the Fermi contact term.

We have used the GIPAW code[10] to model the intersite mixing in $MnBi_2Te_4$ and $MnSb_2Te_4$, and generate the distribution of ^{55}Mn hyperfine fields at the Mn_{3a} and Mn_{6c} nuclei. The calculation details for muon calculations in Supplementary Sec. VI were adopted. Hundreds of different structural configurations were generated in a 2×2×2 supercell of the magnetic primitive cell, according to typical values of the Mn/Bi occupancies for the Wyckoff positions $6c$ (94% Bi and 6%Mn) and $3a$ (74% Mn, 21% Bi and 5% voids) [2], by making use of a supercell code [11]. Of all the generated configurations, one hundred were randomly selected for the DFT calculations of the hyperfine field, involving a total of 800 distinct Mn sites. The resulting distributions of hyperfine fields are shown in Fig.S.9.

V. ZF μSR FITS

The quality of the fits by Eq. 2 can be better judged on representative sets in Fig. S.10. For $MnBi_2Te_4$ at $T = 5$ K the fast initial damping is due to component 1, the visible oscillation is due to component 3, while the contribution of component 2, not so evident in the total best fit, is highlighted by the dashed red curve, where best fit components 1 and 3 have been set to zero amplitude. For $MnBi_6Te_{10}$ at $T = 3$ K component 1 is missing, component 2 is the large amplitude lower frequency oscillation and component 3 is the lower amplitude fast initial oscillation. Figure S.11 shows that the same good fit quality is obtained at all temperatures by Eq. 2 also for $MnBi_2Te_4$ α ($MnBi_2Te_4$-β is in Fig. 2 a).

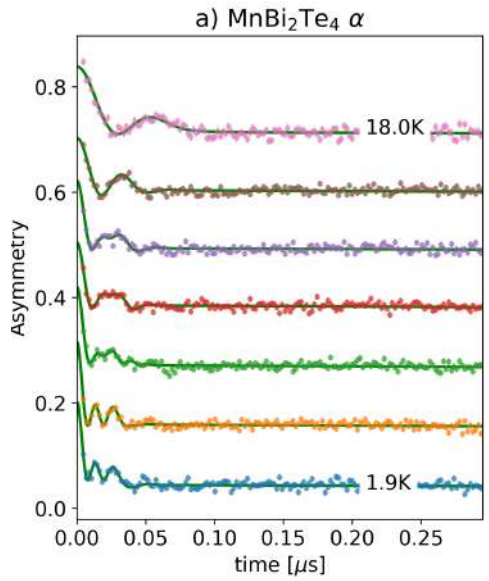

FIG. S.11: Best fits to Eq. 2, at several temperatures for $MnBi_2Te_4$ α sample.

VI. DFT CALCULATION OF THE MUON IMPLANTATION SITE AND COUPLING

A. Methods

We determine the muon implantation sites in $MnBi_2Te_4$ and $MnSb_2Te_4$ by performing collinear spin-polarized density functional theory (DFT) calculations by means of the Quantum Espresso code [12]. We use the projector augmented pseudopotentials [13] and the semi-local generalized-gradient-approximation (GGA) with the Perdew-Burke-Ernzerhof (PBE) [14] functional and the DFT-D3 van der Waals dispersion energy-correction method [15] to capture more accurately the effects of the long-range interactions in the structural optimization. The role of electron-electron interaction on the Mn$-d$ orbitals are considered within the DFT+U scheme [16–19], where we have used a value of $U_{\mathrm{eff}} = (U - J) = 4.0$ eV [20].

The DFT+U relaxations were performed in an inter-layer antiferromagnetic cell (primitive cell doubled along c) for $MnBi_2Te_4$[21]. To accommodate the effect of the spurious artificial interactions of the positive muon impurity modeled with a hydrogen pseudopotential, we have used a 2×2×1 supercell, positively charged, with a uniform charge background to restore charge neutrality. The plane-wave kinetic energy and charge density cut-offs used are 70 Ry and 630 Ry, respectively. For structural optimizations, a 3×3×1 k-point mesh was used for the 57-atom supercell, while it was doubled along all the axes for the calculation of the contact hyperfine field via a self-consistent calculation. The atomic positions were optimized till force and energy thresholds of $1{\times}10^{-3}$ a.u(Ry/Bohr) and $1{\times}10^{-4}$ Ry while the lattice parameters remain fixed at the DFT+U obtained values (i.e., without hydrogen).

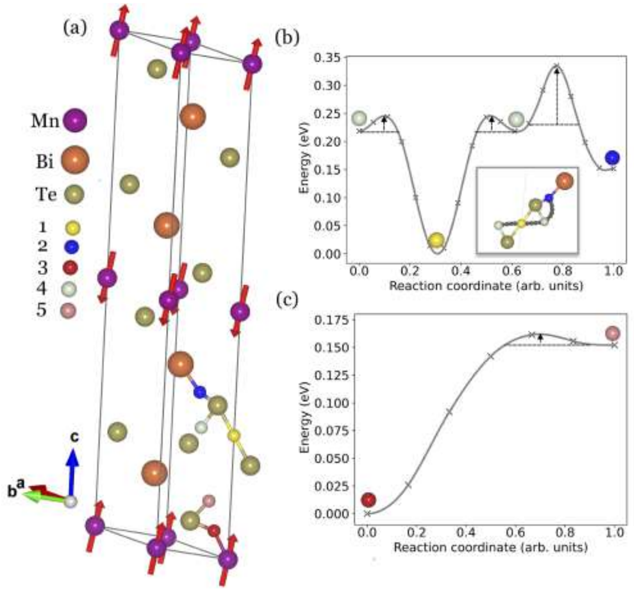

FIG. S.12: (a) Muon sites 1-5 shown in the primitive magnetic cell of $MnBi_2Te_4$(calculations were performed in a 2×2×1 supercell). Nudge elastic band (NEB) minimum energy landscape along two paths connecting calculated muon sites (b) polyline 4′-1-4-2 (site 1 is a high symmetry position and is flanked by sites 4 and its symmetry equivalent, 4′, Inset shows the structural path, where black spheres represents sites between the NEB path), and (c) straight 3-5.

TABLE S.II: DFT relaxed fractional coordinates in the primitive magnetic unit cell of $MnBi_2Te_4$ both for the atomic positions (in half of the magnetic cell) and the calculated muon sites, labelled 1 to 5. The lattice parameters are as follows: $a = b = 4.3795$ Å, $c = 27.6430$ Å, $\alpha = \beta = 80.8841°$, $\gamma = 60.0°$.

Atom	Coordinates
Mn	(0.0, 0.0, 0.0)
Bi	(0.4233, 0.4233, 0.3651)
Bi	(0.5767, 0.5767, 0.1349),
Te(1)	(0.1321, 0.1321, 0.3018)
Te(1)	(0.8679, 0.8679, 0.1982)
Te(2)	(0.2946, 0.2946, 0.0582)
Te(2)	(0.7055, 0.7055, 0.4418)
μ site	Coordinates
1	(0.0000, 0.0001, 0.2499)
2	(0.2697, 0.2697, 0.3191)
3	(0.1134, 0.1134, 0.0499)
4	(0.2461, 0.2461, 0.2495)
5	(0.1333, 0.1884, 0.1077)

TABLE S.III: $MnBi_2Te_4$: muon bond labels and muon sites 1-5 ($\overline{3,5}$ represents the average of 3, 5), DFT total energy difference (ΔE) to the lowest energy and local fields in mT; B_c contact (along the bulk magnetization), B_{dip} dipolar, and B_μ total fields in mT. All fields are rounded to the mT. Magnetic moments on Mn are aligned along the Cartesian z axis ([0001] direction) [22] in the primitive cell. Top section: $MnBi_2Te_4$ muon sites without Mn-Bi intermixing; bottom section: same muon sites affected by the intermixing.

Label	index	ΔE (meV)	B_c	$\mathbf{B}_{dip}$	B_μ
	1	0	0	(0, 0, 0)	0
	4	220	-152	(0, 0, -1)	153
	$4'$[a]	220	152	(0, 0, 1)	153
Te-μ-Te	$\overline{4,1,4'}$		0	(0, 0, 0)	0
Te-μ-Bi	2	150	92	(2, 1, 0)	93
	3	200	-497	(463, 267, -86)	791
	5	350	394	(42, 23, -44)	354
Te-μ-Mn	$\overline{3,5}$		-52	(253, 145, -65)	314

With antisite-mixing

Label	index	nn antisite	B_c	$\mathbf{B}_{dip}$	B_μ
Te-μ-Mn@Bi	2	Mn_{6c}	-282	(443, 256, 156)	527
Te-μ-Bi@Mn	$\overline{3,5}$	Bi_{3a}	236	(-62, -44, -293)	95
Te-μ-Mn($+n.n$ Mn@Bi)[b]	$\overline{3,5}$	Mn_{6c}	288	(-2, -64, -8)	287

[a] Index $4'$ is the symmetry equivalent site of index 4 illustrated in the NEB path, Fig.S.12b.
[b] These are theTe-μ-Mn sites with the closest Bi_{6c} replaced by Mn_{6c} (see Fig S.13e).

B. $MnBi_2Te_4$ muon sites

We start considering $MnBi_2Te_4$ without site intermixing. Structural optimization including accurate treatment of the van der Waals interactions produce five low total energy candidate muon positions, indexed 1, 2, 3, 4 and 5 in Table S.II and Fig. S.12. Muon site 1 has the lowest total DFT energy, taken as the origin of the energy scale. The reported total DFT energy of the other sites are actually the difference ΔE with this reference energy. They are listed in the upper section of Table S.III, together with the local field contributions at each site.

All sites have energies within 0.35 eV, suggesting that all of them are likely to be occupied, although a low energy barrier between close enough sites, or a flat potential landscape may lead to partial delocalization, i.e. the muon wave function could be broad, spreading over the flat potential region. To investigate the stability of these candidate positions, we have performed nudged elastic band (NEB) [23, 24] calculation, mapping the energy landscape along straight muon paths or polylines across close sites (typically, less than 2 Å apart).

The results are shown in Fig. S.12 c for straight path 3-5. Considering that the zero point energy of muons is typically $E_{ZP} \approx 0.5$ eV, the much smaller barriers in the

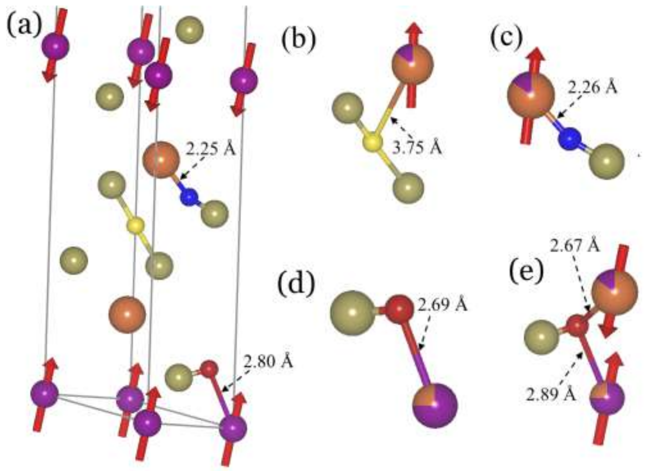

FIG. S.13: Muon sites in $MnBi_2Te_4$ (a) labelled Te-μ-Te (yellow sphere), Te-μ-Bi (blue sphere) and Te-μ-Mn (red sphere). The Mn (at 6c) and Bi (at 3a) site mixing has been considered for (b) the next nearest 6c site of Te-μ-Te, (c) the 6c site of Te-μ-Bi (labelled Te-μ-Mn@Bi), (d) the 3a site of Te-μ-Mn (labelled Te-μ-Bi@Mn), and (e) the next nearest 6c site of Te-μ-Mn (labelled Te-μ-Mn($+n.n$ Mn@Bi)). The value of the μ-Mn/Bi distance is also shown.

energy landscape shows that muons must partially delocalize over both sites 3 and 5, experiencing an average interaction. Similar occurrence happens for the minimum energy path (MEP) of 4′-1-4-2, S.12 b, where from the energy landscape, two distinct muon sites are observed, one among the 4′-1-4 region and the other at site 2. The small energy barrier between 4 and 1 and between the symmetric equivalent 1 and 4′, indicates partial delocalization of the muon over 4′-1-4.

In the following analysis we consider only three main muon sites, labelled Te-μ-Te (delocalized over sites 1, 4 and 4′), Te-μ-Bi (site index 2) and Te-μ-Mn (delocalized over sites 3 and 5) according to their chemical bonds. However, the $MnBi_2Te_4$ sample is characterized by the presence of Bi_{3a} and Mn_{6c} antisites. This generates a large number of local configurations. Guided by experiments we approximate it distinguishing three components with widths smaller than the separation of their means, determined in first approximation by the presence or absence of a nn antisite.

Therefore, we additionally consider the effect of nearest neighbor (nn) antisites on Te-μ-Bi and Te-μ-Mn muon sites. They are listed in the second section of Tab. S.III as Te-μ-Mn@Bi (where we consider the Te-μ-Bi site and Bi_{6c} is replaced by Mn_{6c}), Te-μ-Bi@Mn (where we consider the Te-μ-Mn site and Mn_{3a} is replaced by Bi_{3a}) and Te-μ-Mn($+n.n$ Mn@Bi) (where we consider the Te-μ-Mn site and the closest Bi_{6c} is replaced by Mn_{6c}). The next 3a or 6c site in Te-μ-Te has a distance above 3.5 Angstrom and the small effect on its local field is neglected, in first approximation. These structures and resulting bond distances are show in Figs. S.13 b-e.

C. Local fields in $MnBi_2Te_4$

In a ZF μSR measurement, the total local magnetic field at the muon site consists of the following contributions: $\mathbf{B}_\mu = \mathbf{B}_\mathrm{C} + \mathbf{B}_\mathrm{dip} + \mathbf{B}_\mathrm{L}$, [25] where $\mathbf{B}_\mathrm{c}$ is the isotropic contact contribution originating from the Fermi interaction, which requires a quantum treatment of the electronic wavefunction and has been obtained here with DFT calculations [26]. The last two terms are obtained by the summation of the long-range dipoles in real space using the Lorentz sphere approach, where $\mathbf{B}_\mathrm{dip}$ is the dipolar field obtained from the contributions of the Mn magnetic moments within the sphere, while those outside the sphere contribute to the Lorentz term $\mathbf{B}_\mathrm{L}$ [27], assuming that the outer sample surface is always unmagnetized in ZF. For the antiferromagnetic order of $MnBi_2Te_4$, the $\mathbf{B}_\mathrm{L}$ term vanishes. Table S.III reports our results, where the Mn magnetic moment has been fixed at 4.3 μ_B, the value obtained from our NMR measurements. The non-negligible effect of the muon induced displacements on the host atomic positions is included in calculating B_dip. Due to the approximations inherent in DFT+μ, absolute agreement with experimental values is not expected to be better than within 25%.

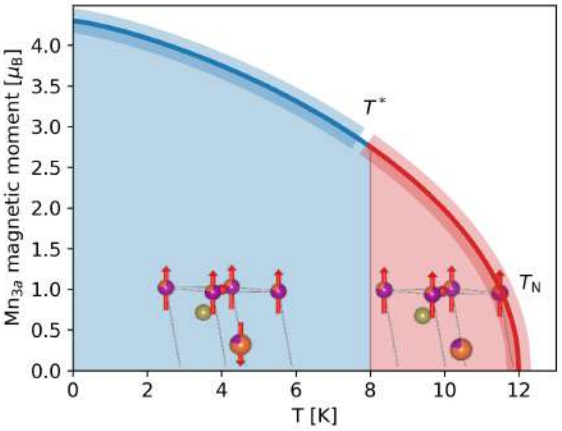

FIG. S.14: $MnBi_6Te_{10}$ temperature dependence of the magnetic moment on Mn_{3a}, low temperature value from NMR, temperature dependence from μSR, $\Delta B_2(T)$. The inset shows the smallest unit cell portion sufficient to highlight the change.

Since the energy minima of sites 3 and 5 are rather close, compared to E_ZP, a rough estimate of their local field is just the value, B_μ, obtained from the average of their local components, reported in Tab. S.III as $\overline{3,5}$. This value is actually in close agreement with the experimental field B_2 at zero temperature.

For more than one site, namely, Te-μ-Te, Te-μ-Bi and Te-μ-Bi@Mn, the predicted local field value is close to zero (within the second moment of the field for component 1, ΔB_1).

Most notable is Te-μ-Mn@Bi, where, due to the short μ-Mn_{6c} distance $\approx$ 2.3 Å, (Fig. S.13c) the internal muon field increases considerably to 527 mT (see Table S.III). This value agrees very closely with the experimental value of B_3 at zero temperature. The site assignment for $MnBi_2Te_4$ is summarized in Tab. I.

D. Local fields in $MnBi_2Te_4 \cdot n(Bi_2Te_3)$, $n = 1, 2$

The $MnBi_2Te_4 \cdot n(Bi_2Te_3)$ structure of the two compounds, is formed by quintuple Bi_2Te_3 layers intercalated between the septuple layers of $MnBi_2Te_4$, therefore we consider the same sites of Tab. S.III, plus further muon sites, similar to Te-μ-Bi and Te-μ-Mn, in the additional quintuple layers. In the FM structure the hyperfine field is never vanishing by symmetry at any site, as it is the case for Te-μ-Te in the AFM structure. Still, most of these site are far away from the Mn_{3a} layer and have small local fields. Therefore they contribute to a broad field distribution centered at the Lorentz field value, $B_\mathrm{L}\approx$ 40 mT (30 mT) for $MnBi_4Te_7$ ($MnBi_6Te_{10}$). This corresponds closely to the experimental B_2 value at zero temperature. Its temperature dependence is shown in Fig. S.14 for $n = 2$, rescaled to the magnetic moment

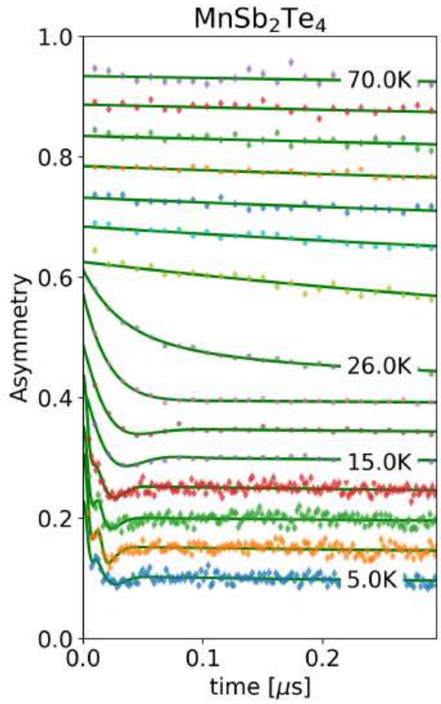

FIG. S.15: $MnSb_2Te_4$ μSR asymmetry with best fit to Eq. 2 (see text).

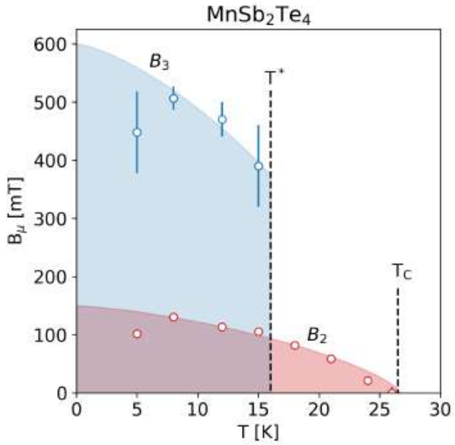

FIG. S.16: Best fit fields B_2 and B_3 in $MnSb_2Te_4$.

per site with the ZF NMR calibration at 1.4 K, and T^* is the temperature where the B_3 term disappears (see Fig. 2 f).

The field value corresponding to site Te-μ-Mn, the highest energy site in $MnBi_2Te_4$, is not observed experimentally. This is not surprising, in view both of its higher energy and of the large number of sites contributing to B_2.

E. Local fields in $MnSb_2Te_4$

Figure S.15 shows the best fits to Eq. 2, where the overdamped B_1=0 transverse component is missing, and Fig. S.16 shows the best-fit field values vs. temperature.

The calculation yields similar results and the muon site classification is similar to that of $MnBi_2Te_4$, but the actual sample interlayer order is ferromagnetic,

giving rise to a Lorentz contribution $B_L \approx 70$ mT. Here, the difference between the Sb and the Mn radii is even smaller than in the Bi case, therefore the occupancy of the Mn_{6c} antisite is larger, and all local field distributions become much broader. The B_2 component agrees with the prediction for all the sites that have low local field values in $MnBi_2Te_4$, namely, Te-μ-Te , Te-μ-Bi and Te-μ-Bi@Mn.

[1] V. Petříček, M. Dušek, and L. Palatinus, Crystallographic computing system jana2006: General features, Z. Kristallogr. Cryst. Mater **229**, 345 (2014).

[2] A. Zeugner, F. Nietschke, A. U. Wolter, S. Gaß, R. C. Vidal, T. R. Peixoto, D. Pohl, C. Damm, A. Lubk, R. Hentrich, *et al.*, Chemical aspects of the candidate antiferromagnetic topological insulator mnbi2te4, Chemistry of Materials **31**, 2795 (2019).

[3] D. Souchay, M. Nentwig, D. Günther, S. Keilholz, J. de Boor, A. Zeugner, A. Isaeva, M. Ruck, A. U. Wolter, B. Büchner, *et al.*, Layered manganese bismuth tellurides with gebi 4 te 7-and gebi 6 te 10-type structures: towards multifunctional materials, Journal of Materials Chemistry C **7**, 9939 (2019).

[4] L. C. Folkers, L. T. Corredor, F. Lukas, M. Sahoo, A. U. Wolter, and A. Isaeva, Occupancy disorder in the magnetic topological insulator candidate mn1- x sb2+ x te4, Zeitschrift für Kristallographie-Crystalline Materials **237**, 101 (2022).

[5] M. Sahoo, Z. Salman, G. Allodi, A. Isaeva, L. Folkers, A. Wolter, B. Büchner, and R. De Renzi, Impact of mn-pn intermixing on magnetic properties of an intrinsic magnetic topological insulator: the μsr perspective, in *Journal of Physics: Conference Series*, Vol. 2462 (IOP Publishing, 2023) p. 012040.

[6] S. H. Lee, Y. Zhu, Y. Wang, L. Miao, T. Pillsbury,

H. Yi, S. Kempinger, J. Hu, C. A. Heikes, P. Quarterman, W. Ratcliff, J. A. Borchers, H. Zhang, X. Ke, D. Graf, N. Alem, C.-Z. Chang, N. Samarth, and Z. Mao, Spin scattering and noncollinear spin structure-induced intrinsic anomalous hall effect in antiferromagnetic topological insulator $MnBi_2Te_4$, Phys. Rev. Res. **1**, 012011 (2019).

[7] S.-K. Bac, K. Koller, F. Lux, J. Wang, L. Riney, K. Borisiak, W. Powers, M. Zhukovskyi, T. Orlova, M. Dobrowolska, *et al.*, Topological response of the anomalous hall effect in mnbi2te4 due to magnetic canting, npj quantum materials **7**, 46 (2022).

[8] P. M. Sass, W. Ge, J. Yan, D. Obeysekera, J. Yang, and W. Wu, Magnetic imaging of domain walls in the antiferromagnetic topological insulator mnbi2te4, Nano letters **20**, 2609 (2020).

[9] The Elk Code, `http://elk.sourceforge.net/`.

[10] C. J. Pickard and F. Mauri, All-electron magnetic response with pseudopotentials: Nmr chemical shifts, Phys. Rev. B **63**, 245101 (2001).

[11] K. Okhotnikov, T. Charpentier, and S. Cadars, Supercell program: a combinatorial structure-generation approach for the local-level modeling of atomic substitutions and partial occupancies in crystals, Journal of Cheminformatics **8**, 17 (2016).

[12] P. Giannozzi, S. Baroni, N. Bonini, M. Calandra, R. Car, C. Cavazzoni, D. Ceresoli, G. L. Chiarotti, M. Cococcioni, I. Dabo, A. Dal Corso, S. de Gironcoli, S. Fabris, G. Fratesi, R. Gebauer, U. Gerstmann, C. Gougoussis, A. Kokalj, M. Lazzeri, L. Martin-Samos, N. Marzari, F. Mauri, R. Mazzarello, S. Paolini, A. Pasquarello, L. Paulatto, C. Sbraccia, S. Scandolo, G. Sclauzero, A. P. Seitsonen, A. Smogunov, P. Umari, and R. M. Wentzcovitch, QUANTUM ESPRESSO: a modular and open-source software project for quantum simulations of materials, Journal of Physics: Condensed Matter **21**, 395502 (19pp) (2009).

[13] P. E. Blöchl, Projector augmented-wave method, Phys. Rev. B **50**, 17953 (1994).

[14] J. P. Perdew, K. Burke, and M. Ernzerhof, Generalized gradient approximation made simple, Phys. Rev. Lett. **77**, 3865 (1996).

[15] S. Grimme, J. Antony, S. Ehrlich, and H. Krieg., A consistent and accurate ab initio parametrization of density functional dispersion correction (dft-d) for the 94 elements h-pu, The Journal of Chemical Physics **132**, 154104 (2010).

[16] S. L. Dudarev, G. A. Botton, S. Y. Savrasov, C. J. Humphreys, and A. P. Sutton, Electron-energy-loss spectra and the structural stability of nickel oxide: An lsda+u study, Phys. Rev. B **57**, 1505 (1998).

[17] M. Cococcioni and S. de Gironcoli, Linear response approach to the calculation of the effective interaction parameters in the LDA + U method, Phys. Rev. B **71**, 035105 (2005).

[18] H. J. Kulik, M. Cococcioni, D. A. Scherlis, and N. Marzari, Density Functional Theory in Transition-Metal Chemistry: A Self-Consistent Hubbard U Approach, Phys. Rev. Lett. **97**, 103001 (2006).

[19] I. Timrov, N. Marzari, and M. Cococcioni, Hubbard parameters from density-functional perturbation theory, Phys. Rev. B **98**, 085127 (2018).

[20] P. Li, J. Yu, Y. Wang, and W. Luo, Electronic structure and topological phases of the magnetic layered materials $mnbi_2te_4, mnbi_2se_4$, and $mnsb_2te_4$, Phys. Rev. B **103**, 155118 (2021).

[21] M. M. Otrokov, I. I. Klimovskikh, H. Bentmann, D. Estyunin, A. Zeugner, Z. S. Aliev, S. Gaß, A. U. B. Wolter, A. V. Koroleva, A. M. Shikin, M. Blanco-Rey, M. Hoffmann, I. P. Rusinov, A. Y. Vyazovskaya, S. V. Eremeev, Y. M. Koroteev, V. M. Kuznetsov, F. Freyse, J. Sánchez-Barriga, I. R. Amiraslanov, M. B. Babanly, N. T. Mamedov, N. A. Abdullayev, V. N. Zverev, A. Alfonsov, V. Kataev, B. Büchner, E. F. Schwier, S. Kumar, A. Kimura, L. Petaccia, G. Di Santo, R. C. Vidal, S. Schatz, K. Kißner, M. Ünzelmann, C. H. Min, S. Moser, T. R. F. Peixoto, F. Reinert, A. Ernst, P. M. Echenique, A. Isaeva, and E. V. Chulkov, Prediction and observation of an antiferromagnetic topological insulator, Nature **576**, 416 (2019).

[22] J.-Q. Yan, Q. Zhang, T. Heitmann, Z. Huang, K. Y. Chen, J.-G. Cheng, W. Wu, D. Vaknin, B. C. Sales, and R. J. McQueeney, Crystal growth and magnetic structure of $MnBi_2Te_4$, Phys. Rev. Mater. **3**, 064202 (2019).

[23] H. Jónsson, G. Mills, and K. W. Jacobsen, Nudged elastic band method for finding minimum energy paths of transitions, in *Classical and Quantum Dynamics in Condensed Phase Simulations*, edited by B. J. Berne, G. Ciccotti, and D. F. Coker (World Scientific, 1998).

[24] G. Henkelman, B. P. Uberuaga, and H. Jø'nsson, A climbing image nudged elastic band method for finding saddle points and minimum energy paths, The Journal of Chemical Physics **113**, 9901 (2000), https://pubs.aip.org/aip/jcp/article-pdf/113/22/9901/10828159/9901_1_online.pdf.

[25] S. J. Blundell, R. De Renzi, T. Lancaster, and F. L. e. Pratt, *Introduction to Muon Spectroscopy* (Oxford University Press, Oxford, 2022).

[26] I. J. Onuorah, P. Bonfà, and R. De Renzi, Muon contact hyperfine field in metals: A dft calculation, Phys. Rev. B **97**, 174414 (2018).

[27] P. Bonfà, I. J. Onuorah, and R. D. Renzi, Introduction and a quick look at muesr, the magnetic structure and muon embedding site refinement suite, in *Proceedings of the 14th International Conference on Muon Spin Rotation, Relaxation and Resonance (μSR2017)* (the Physical Society of Japan, 2018) https://journals.jps.jp/doi/pdf/10.7566/JPSCP.21.011052.
